# Enabling High Precision Gradient Index Control in Subsurface Multiphoton Lithography

**DOI:** 10.1021/acsphotonics.2c01950

**Published:** 2023-03-29

**Authors:** Alexander J. Littlefield, Dajie Xie, Corey A. Richards, Christian R. Ocier, Haibo Gao, Jonah F. Messinger, Lawrence Ju, Jingxing Gao, Lonna Edwards, Paul V. Braun, Lynford L. Goddard

**Affiliations:** † Department of Electrical and Computer Engineering, University of Illinois Urbana-Champaign, Urbana, Illinois 61801, United States; ‡ Nick Holonyak, Jr., Micro and Nanotechnology Laboratory, University of Illinois Urbana-Champaign, Urbana, Illinois 61801, United States; § Department of Materials Science and Engineering, University of Illinois Urbana-Champaign, Urbana, Illinois 61801, United States; ∥ Materials Research Laboratory, University of Illinois Urbana-Champaign, Urbana, Illinois 61801, United States; ⊥ Beckman Institute for Advanced Science and Technology, University of Illinois Urbana-Champaign, Urbana, Illinois 61801, United States; # Department of Physics, University of Illinois Urbana-Champaign, Urbana, Illinois 61801, United States; ∇ Department of Mechanical Science and Engineering, University of Illinois Urbana-Champaign, Urbana, Illinois 61801, United States

**Keywords:** 3D direct laser writing, multiphoton microscopy, graded index lens, high contrast gratings, porous silicon

## Abstract

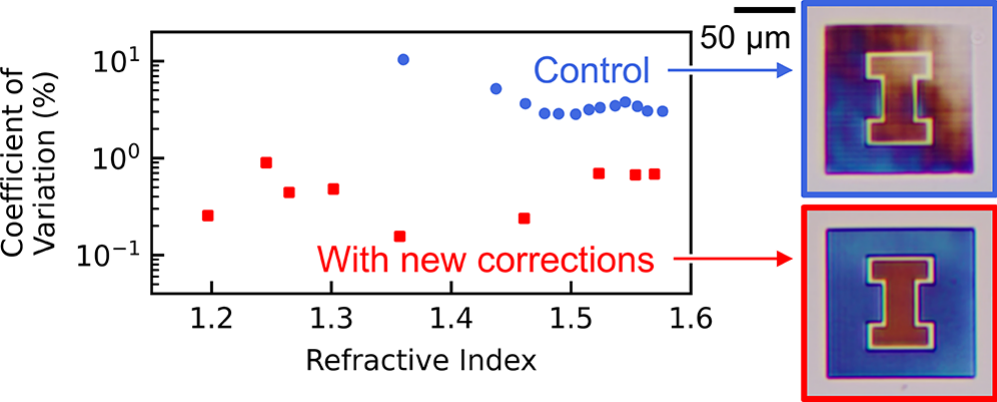

Multiphoton lithography inside a mesoporous host can create optical components with continuously tunable refractive indices in three-dimensional (3D) space. However, the process is very sensitive at exposure doses near the photoresist threshold, leading previous work to reliably achieve only a fraction of the available refractive index range for a given material system. Here, we present a method for greatly enhancing the uniformity of the subsurface micro-optics, increasing the reliable index range from 0.12 (in prior work) to 0.37 and decreasing the standard deviation (SD) at threshold from 0.13 to 0.0021. Three modifications to the previous method enable higher uniformity in all three spatial dimensions: (1) calibrating the planar write field of mirror galvanometers using a spatially varying optical transmission function which corrects for large-scale optical aberrations; (2) periodically relocating the piezoelectrically driven stage, termed piezo-galvo dithering, to reduce small-scale errors in writing; and (3) enforcing a constant time between each lateral cross section to reduce variation across all writing depths. With this new method, accurate fabrication of optics of any index between *n* = 1.20 and 1.57 (SD < 0.012 across the full range) was achieved inside a volume of porous silica. We demonstrate the importance of this increased accuracy and precision by fabricating and characterizing calibrated two-dimensional (2D) line gratings and flat gradient index lenses with significantly better performance than the corresponding control devices. As a visual representation, the University of Illinois logo made with 2D line gratings shows significant improvement in its color uniformity across its width.

## Introduction

Direct laser writing (DLW) is a versatile fabrication method for making a broad variety of devices including micromechanical structures, microfluidics, waveguides, and custom-designed lenses.^1−6^ Recent developments have allowed for subsurface gradient refractive index (GRIN) devices to be written within a three-dimensional volume of a host material using a direct laser writing method we term subsurface controllable refractive index via beam exposure (SCRIBE).^7^ In this method, a mesoporous medium is first infilled with photoresist. Then, by focusing a femtosecond laser into the volume of the infilled host material and modulating the laser power as a function of position, a variable density of photoresist can be cross-linked in localized regions throughout the volume of the medium. The variable density of polymerized resist gives rise to a continuously variable GRIN profile. Specifically, this process has been shown using IP-Dip (distributed by Nanoscribe GmbH) as the photoresist and the Nanoscribe Photonic Professional GT as the DLW instrument.

**Figure 1:**
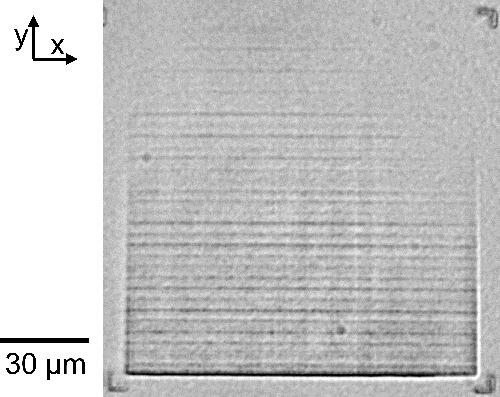
Rectangular prism (*L* = 120 μm × *W* = 120 μm × *H* = 5 μm) imaged under a standard bright-field microscope. Although the average exposure power was set to be uniform at 11.5 mW (1.46 TW/cm^2^ peak intensity) across the entire device, significant fading is visible in the top right corner. Furthermore, repeated unexpected lines appear parallel to the *x*-axis every 3–7 μm even though the laser is scanned in the +*y* direction. Both errors are especially prominent in devices fabricated near the threshold exposure power.

Previous work by others in the literature demonstrates above-surface DLW GRIN without a porous medium, but the refractive index range is typically limited to the order of 0.01.^8,9^ Previously, SCRIBE, when performed in porous silica (abbreviated as PSiO_2_), realized a continuous index range of 0.22 (from *n* = 1.36 to 1.58), with a reliable index range of only 0.12 (from *n* = 1.46 to 1.58), where the reliable index range is defined as having a refractive index standard deviation (SD) of less than 0.05 between identical devices. However, neither of these are close to the theoretical maximum refractive index range of 0.40 (from *n* = 1.15 [empty PSiO_2_] to *n* = 1.55 [maximum infilling of IP-Dip]). These index measurements were collected at a wavelength of 633 nm. When devices are fabricated near the threshold laser power, significant errors appear as shown in Figure 1.

**Figure 2:**
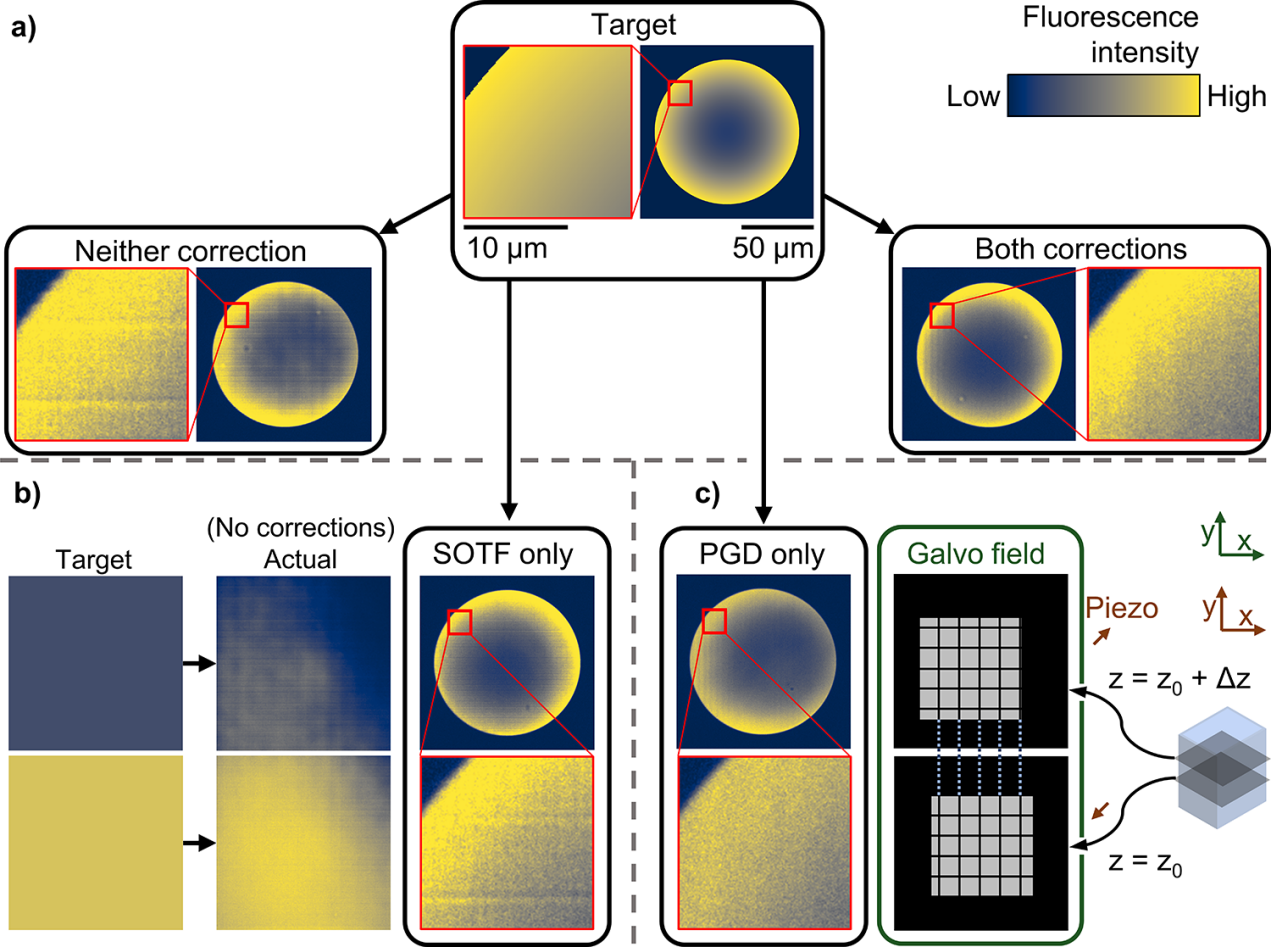
Outline of SOTF and PGD methods and results for improving the fidelity of written structures. The intensity in a fluorescence image is positively correlated with the local refractive index.^7^ (a) Overview: The target fluorescence image for a flat diverging lens is compared with the measured image of a control device (left) and a calibrated device with both SOTF and PGD corrections (right). Large-scale errors (e.g., faded top right corner) and small-scale errors (horizontal lines in the inset) are both present in the control but are reduced or eliminated after applying the full corrections. (b) SOTF method: SOTF begins with collection of calibration data, as shown on the left. The target refractive index is fitted to the calibration data to produce an output laser power that varies spatially to correct large-scale systematic nonuniformities. After applying only SOTF to the lens design, the measured fluorescence of the fabricated device shows that the large-scale errors are minimized but that the small-scale errors remain. (c) PGD method: For each *z* plane, the PGD method moves the piezo in the *xy* plane and compensates with the galvo position such that the actual position of the laser focus on the sample does not change (e.g., we move the piezo by (Δ*x*, Δ*y*) = (+5, +5 μm) and the galvo by (Δ*x*, Δ*y*) = (−5, −5 μm)), as demonstrated on the right. In the PGD-only lens, small-scale errors are smoothed out by periodically relocating the galvo, but the large-scale fading remains. All images in Figure 1 are fabricated with constant-time correction enabled. All fluorescence images use the same 50 μm scale bar, with the exception of insets (shown with a red border), which use a 10 μm scale bar.

In this work, we improve the reproducibility of refractive index by over an order of magnitude via a newly designed calibration process. This calibration process addresses spatial effects using both the spatially varying optical transmission function (SOTF) and piezo-galvo dithering (PGD) methods, as outlined in Figure 2, while temporal effects are addressed using the constant time (CT) correction method. We demonstrate the impact of improved reproducibility on optical devices, including Fresnel biprisms, diffraction gratings, and GRIN lenses. The method requires only one-time collection of calibration data paired with software corrections, and it increases the fabrication time required by less than 10% for structures using the full write field. Furthermore, the causes of the previously low reproducibility can be identified through evaluation of the calibration process. Even in the fully corrected lenses shown in Figure 2, some artifacts remain due to temporal laser power variation (Figure S1), inaccurate fluorescence measurement of high-density polymer (Figure S2), cracks in the porous silica film after polymerization (Figure S3), and defects in the porous silica film or photoresist (Table S1).

**Figure 3:**
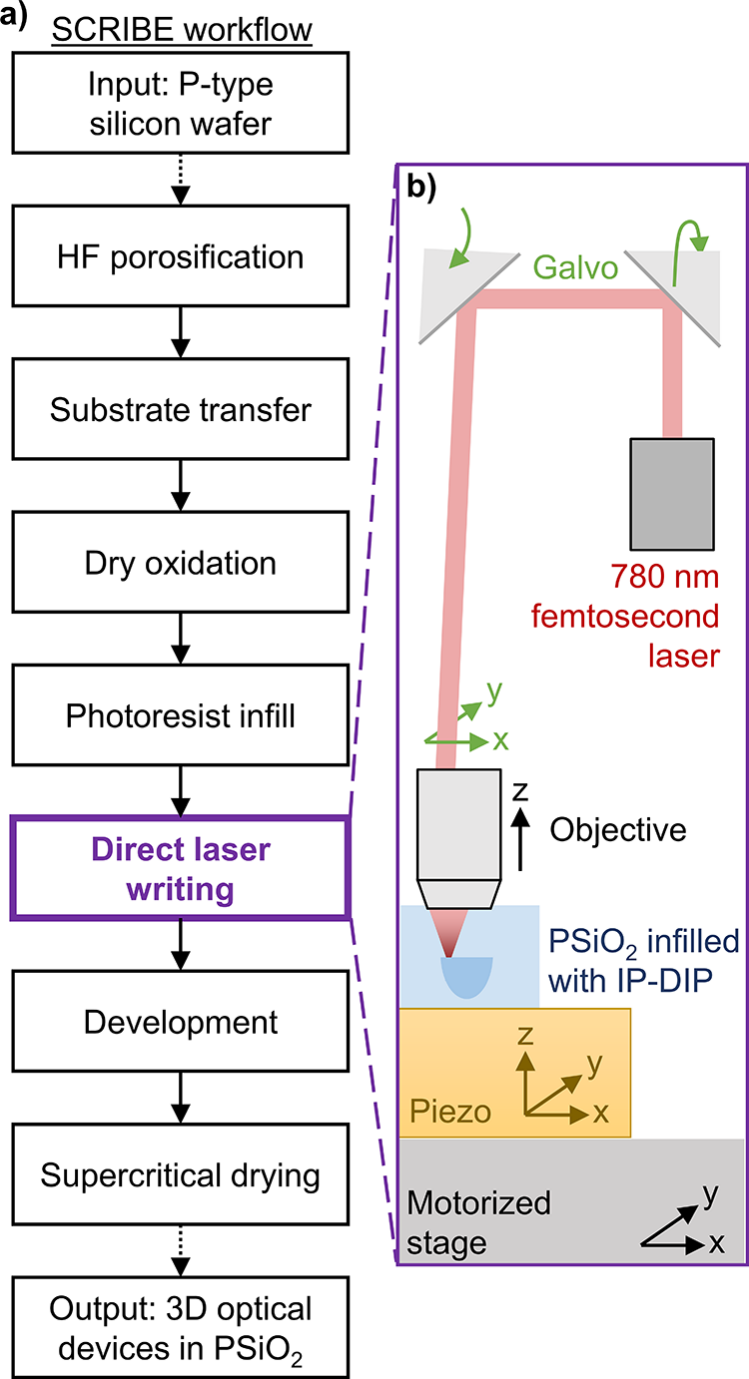
(a) High-level overview of the SCRIBE process workflow, including the raw materials needed and the resulting output. (b) Instrument internals: A simplified diagram of the components inside the DLW instrument (Nanoscribe Photonic Professional GT). This paper focuses exclusively on improving the “direct laser writing” step.

To appreciate the motivation for the improvements presented herein, the positioning systems of the DLW instrument and mechanics of the photoresist must first be understood. The SCRIBE workflow is shown in Figure 3a, and the internals of the DLW instrument used are shown in Figure 3b. Light emitted by the laser source is redirected by two galvanometer *xy*-scanning mirrors (galvo) and focused by the writing objective into the sample volume of PSiO_2_. The objective can move in *z* to set the focus at the sample interface. The piezoelectric *xyz*-translation stage (piezo) precisely moves the sample small distances (<300 μm), while the motorized *xy*-translation stage coarsely moves the sample large distances (up to 20 cm). For our DLW instrument, the typical piezo scan speed is 100 μm/s, limited by the inertia of the sample holder. By contrast, the galvo is capable of moving the laser focus at speeds of over 100,000 μm/s. The galvo is inherently faster due to the smaller distance the mirrors must travel to change the angle of the light,^10^ while the high-resolution piezo must move the full scan distance. Writing with the galvo, or another method by which different angles incident on the objective are used to map to different positions in the system (such as light-sheet microprinting^11^), is necessary to fabricate typical micro-optic structures in a reasonable amount of time.^12^

Previous work in the literature combined multiple different positioning systems to reduce errors when stitching (switching between positioning systems).^13^ In this work, we apply a similar but distinct method for reducing errors within a single write field.

As the galvo sweeps the laser focus across the sample, the laser beam passes through a different part of the objective for each section of the write field, resulting in an aggregation of small changes to optical intensity from field curvature and other aberrations in the optics.^14^ Furthermore, the galvo mirrors have lower precision in maintaining a constant raster velocity (and thus, the total exposure dose) than the piezo because the galvo is driven by traditional electromagnetic motors. Some methods have been developed to mitigate field distortions,^15^ but the local intensity at the focal point still cannot be held perfectly constant. Furthermore, new developments in the field have created piezoelectric-driven galvo mirrors which may be more precise, although these are not yet commercially available.^16^ Quantization error in the galvo positioning is also a source of error.^17^

The photoresist in the mesoporous scaffold is extremely sensitive in the threshold region, changing in refractive index from 1.20 to 1.35 (38% of its range) with a change in average exposure power from 10.8 to 12.0 mW (peak intensities from 1.37 to 1.53 TW/cm^2^; 11% increase). Outside the threshold region, a change in refractive index from 1.46 to 1.57 (28% of its range) requires a change in average exposure power from 13.3 to 16.9 mW (1.69 to 2.15 TW/cm^2^; 27% increase). Due to small systematic errors, the exposure dose is not perfectly uniform across the exposure field, which leads to spatial variation in the local index. Due to the high sensitivity near threshold, even a small exposure intensity variation has a substantial impact on GRIN optics fabricated with SCRIBE. By contrast, constant-index optics fabricated with conventional DLW are not sensitive to moderate variations in exposure dose. Conventional DLW uses exposure doses well above the threshold, a region where the index versus exposure curve is almost flat.^12^ Interference-based devices, e.g., prisms, become effectively unusable when written near the threshold because they are sensitive to small index variations.^7^ Writing artifacts due to this sensitivity appear consistently and can be viewed by multiple microscopes (Figure S5), and several of these errors match well with those found in the literature.^18^

The optical properties of IP-Dip, the photoresist used in this work, have been extensively studied.^19−21^ When used in the standard fabrication mode where polymerization occurs above the surface of the substrate, IP-Dip has been observed to have a variable refractive index range of 0.01.^22^ The increase in refractive index variance previously seen near the threshold of the photoresist is caused by rapidly varying solubility. A logistic function can be used to model the full solubility curve for cross-linked photoresist and explain the rapid variation near the threshold.^23^ Even above-surface fabrication methods enabling GRIN show an increase in refractive index variance at lower laser powers,^9^ similar to that shown in SCRIBE.

Because the polymer is below the surface of the sample for SCRIBE, imaging the full three-dimensional (3D) writing pattern is nontrivial; it is not possible to image the full writing pattern via methods such as scanning electron microscopy (SEM) or atomic force microscopy (AFM), which are typically used to evaluate lithographically patterned structures. While cross sections of structures can be seen in some cases (e.g., after cleaving) via SEM,^7^ even then the images do not provide quantitative density measurements, which are necessary for calibration.^24^ AFM is a surface-only technique, and it cannot easily detect density changes to the resolution needed for calibration.^25^ Although AFM can detect height changes, the height of the polymer does not necessarily correlate with its refractive index. In particular, the changing focus of the objective during writing may cause the refractive index vs polymer height function to change as a function of position. Imaging techniques such as confocal microscopy, bright-field microscopy, or dark-field microscopy do not provide the quantitative information necessary to correct the aberrations.^26^

Two-photon fluorescence microscopy is uniquely suited to collect calibration data on the galvo scan field. It gives sufficient resolution and enables quantitative density measurements of the photoresist within the structure.^27^ Because the porous silica films used in this work are only 20 μm thick, the two-dimensional (2D) profile of the galvo scan field measured at one *z* location should be reasonably close to the 2D profile at other depths within this thin film. Measurement of polymer density is possible at the initial writing wavelength of 780 nm due to the small amount of residual photoinitiator trapped inside the final polymerized structure after development. We previously showed a correlation between fluorescence intensity and refractive index,^7^ and in this work, we show that this correlation is reliable enough to establish a quantitative mapping.

## Concept

To improve the uniformity and accuracy of written devices, a three-part calibration procedure has been developed.

### Constant-Time (CT) Correction

We observed that the threshold for polymerization depends strongly on the micro- and macro-time scales of the writing process. See Figure S6. Therefore, devices written near threshold for which these time scales are not held constant throughout the writing will exhibit large variations in index. We developed a simple fix to address these temporal effects: adjacent voxels are written with constant time between them independent of the 3D geometry. To achieve this within a particular *z* layer, the rest of the 100 × 100 μm^2^ write field—even where polymer is not desired—is written with a below-threshold laser power (0.01% = 5 μW). Enforcing a constant micro-time delay between voxels of a line, constant macro-time delay between adjacent lines in a layer, and constant mega-time delay between each *z* layer increases the uniformity of the polymerization dynamics and therefore reduces undesirable variation of the local 3D refractive index profile. The CT correction is described in more detail in Figure S7. Application of the constant-time correction also improves the uniformity of variable-length 3D photonic waveguides.^28^ Further, the CT correction reduces unintended roughness in waveguides,^28^ which is known to create coherent backscattering and mode splitting in microring resonators.^29^

### Spatially Varying Optical Transmission Function (SOTF)

A mathematical function is created that spatially modulates the laser power such that the desired refractive index is maintained regardless of the spatial location of the laser focus. The first step to achieving this is to acquire an SOTF as outlined in Figure 2b. This function is approximated by a 3D image stack. To simplify the creation of the SOTF, we assume the power output from consecutive pulses of the laser has minimal temporal dependence, which includes both transitory fluctuations and long-term system drift. We confirmed the validity of this assumption with measurements from our DLW instrument (see Figure S1).

**Figure 4:**
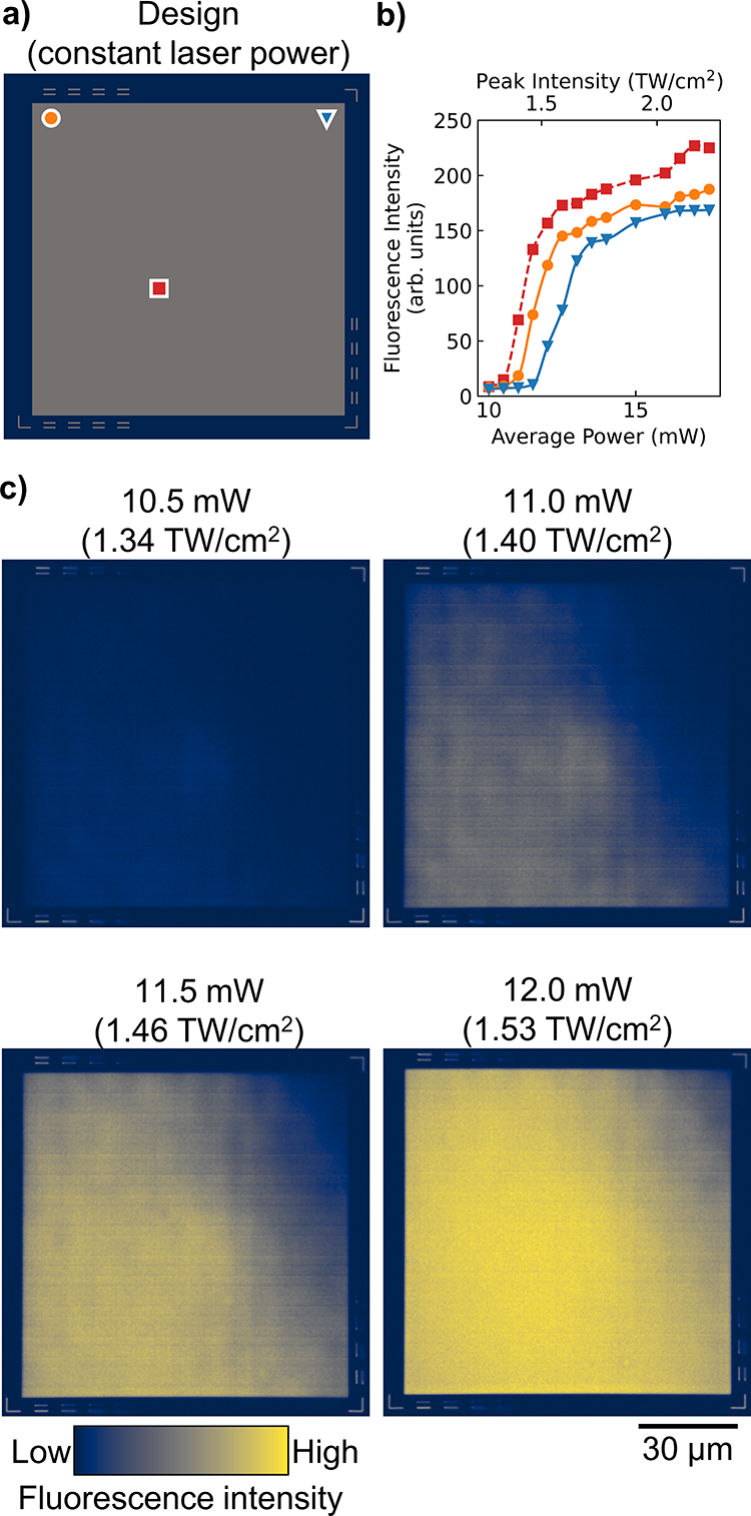
(a) Design of calibration device, with three locations marked. (b) Fluorescence intensity as measured by multiphoton microscopy at locations marked in (a). (c) Images from multiphoton imaging with fabrication at four constant average laser powers, varying linearly from 10.5 mW (top left, peak intensity of 1.34 TW/cm^2^) to 12.0 mW (bottom right, peak intensity of 1.53 TW/cm^2^).

To capture the SOTF, a calibration sample is fabricated and measured as shown in Figure 4. The calibration design, shown in Figure 4a, is composed of: (1) a rectangular prism measuring 120 × 120 × 5 μm^3^ centered in the design field; (2) corner alignment marks composed of two lines each (5 × 1 × 1 μm^3^) at three of the four edges of the horizontal plane; and (3) vertical alignment marks with varying z-spacing. To collect polymer density data, images of these samples were taken using a multiphoton microscope. The microscope focus was centered on the rectangular prism by selecting the *z* plane at which adjacent vertical alignment marks were equally visible, mitigating any extra nonuniformity due to inconsistent imaging planes in *z*. Our custom software can calibrate for both in-plane translation and rotation after imaging because the three alignment marks are at known locations in the writing mask. Identical samples were fabricated at the University of Illinois Chicago to confirm that similar issues exist in other instruments, but the exact patterns were found to be instrument-specific (see Figure S8).

As shown in Figure 4b, the polymerization threshold with respect to input laser power varies laterally across the sample. Each location has a different focal profile due to small aberrations from the objective. These differences have a strong effect near threshold, as can be seen from the imaged calibration devices (Figure 4c). As confirmed by this empirical data, using a constant laser power is insufficient to produce a constant refractive index.

The fluorescence images from Figure 4c are used to construct the SOTF map. First, the image data is binned spatially (1 × 1 μm^2^) to reduce the effect of random noise. When applying SOTF, the nearest bin is used for laser power compensation. Smaller bins of 0.1 × 0.1 μm^2^ were tested but did not improve the resulting devices and significantly increased the fabrication file size. Next, the measured fluorescence intensity vs fabrication laser power is fit to a piecewise cubic Hermite interpolating polynomial (PCHIP) for each bin. A PCHIP fit provides accurate modeling for a complicated underlying function, such as the SOTF here.^30^ Then, a specific fluorescence intensity is selected as the target polymer density for each location within the designed device. The laser power at each bin is then back-calculated via the PCHIP fit. Finally, for several constant target polymer densities, Fresnel biprisms are written with the back-calculated laser power to establish the mapping between fluorescence intensity and refractive index, as determined from the measured interference pattern of the biprisms. Thus, a concrete mapping between output refractive index and input laser power can be determined as a function of lateral position.

Mathematically, we define the SOTF as a time-independent empirical function with inputs including the lateral position of the galvo (*x*′, *y*′) and the target refractive index (*n*) and whose output is the laser power (*L*), as shown in eq 1.
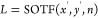
1When a given refractive index is desired that was not explicitly measured during the calibration, an interpolated laser power may be numerically computed to determine the SOTF. To disable the SOTF functionality (e.g., for the control devices), the SOTF is averaged over all positions, allowing a constant laser power *L̅*(*n*) to be used. Additionally, above a certain refractive index (approximately *n* = 1.35 in this experiment), the SOTF performs worse than the control (see Figure S2). Thus, to obtain the best overall correction, the final laser power is given by eq 1 for *n* < 1.35, *L̅* for *n* > 1.40, and a linear interpolation of these two functions for indices in between.

We hypothesize the main causes of large-scale aberrations are field curvature and other forms of imaging aberrations of the writing instrument. Assuming the laser writing system characteristics do not change after calibration, the SOTF correction can properly set the laser power to achieve the desired refractive index anywhere within the write field. After performing the SOTF calibration, uniformity in the refractive index over the write field is improved, but significant small-scale inhomogeneities remained, motivating a further correction.

### Piezo-Galvo Dithering (PGD)

Although the SOTF correction eliminates large-scale errors, some small-scale errors cannot be compensated for by changing laser power alone. A method that we term PGD is implemented to solve these remaining errors. At each voxel location, PGD changes the absolute position of the galvo while simultaneously moving the piezo such that the physical location in the sample at which polymerization occurs remains unchanged. A high-level overview of PGD is shown in Figure 2c.

First, the piezo is stepped in increments of +1 μm in each lateral direction on each *z* layer, i.e., the motion is diagonal. When the piezo reaches the end of its scan (set to +7 μm), the piezo location is reset to its original position of −7 μm. At the same time, the galvo is repositioned appropriately such that the actual writing location will be that of the design file. The combination of both piezo and galvo movement allows for effectively physically averaging out the small-scale artifacts that exist in galvo-only writing by oversampling vertically. The height polymerized by the laser focus is approximately 1 μm, and the gap between subsequent *xy* scan planes is 0.1 μm. Therefore, several adjacent layers, each with slightly different small-scale errors, are averaged together to improve overall uniformity.

With PGD, both horizontal and vertical bands (small-scale errors) are appreciably reduced with only a 7% decrease in writing speed (time needed for the piezo to settle). The decrease in writing speed corresponds to one extra second of piezo settling time per layer of voxels. PGD is limited to removing small-scale aberrations relative to the size of the write field because every micron used by PGD is one micron removed from the available write field for the device.

Mathematically, we define PGD to be a function whose inputs are the physical coordinates of the desired voxel (*x*, *y*, *z*) and three adjustable parameters, *i*, *j*, and *k*, and whose outputs are the galvo coordinates (*x*′, *y*′), as shown in eqs 2.a and 2.b.
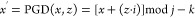
2.a
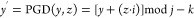
2.bThe ideal choice for the values of *i*, *j*, and *k* depends on the spacing between layers and the size of the laser’s focus. Generally, we found *i* = 10, *j* = 15 μm, and *k* = 7 μm to work well on our DLW instrument, which bounds the function between −7 and +7 μm from the current position. The PGD function will step 1 μm laterally for each layer in *z*, which has a separation of 0.1 μm in our implementation.

## Results and Discussion

### Rectangular Prisms

**Figure 5:**
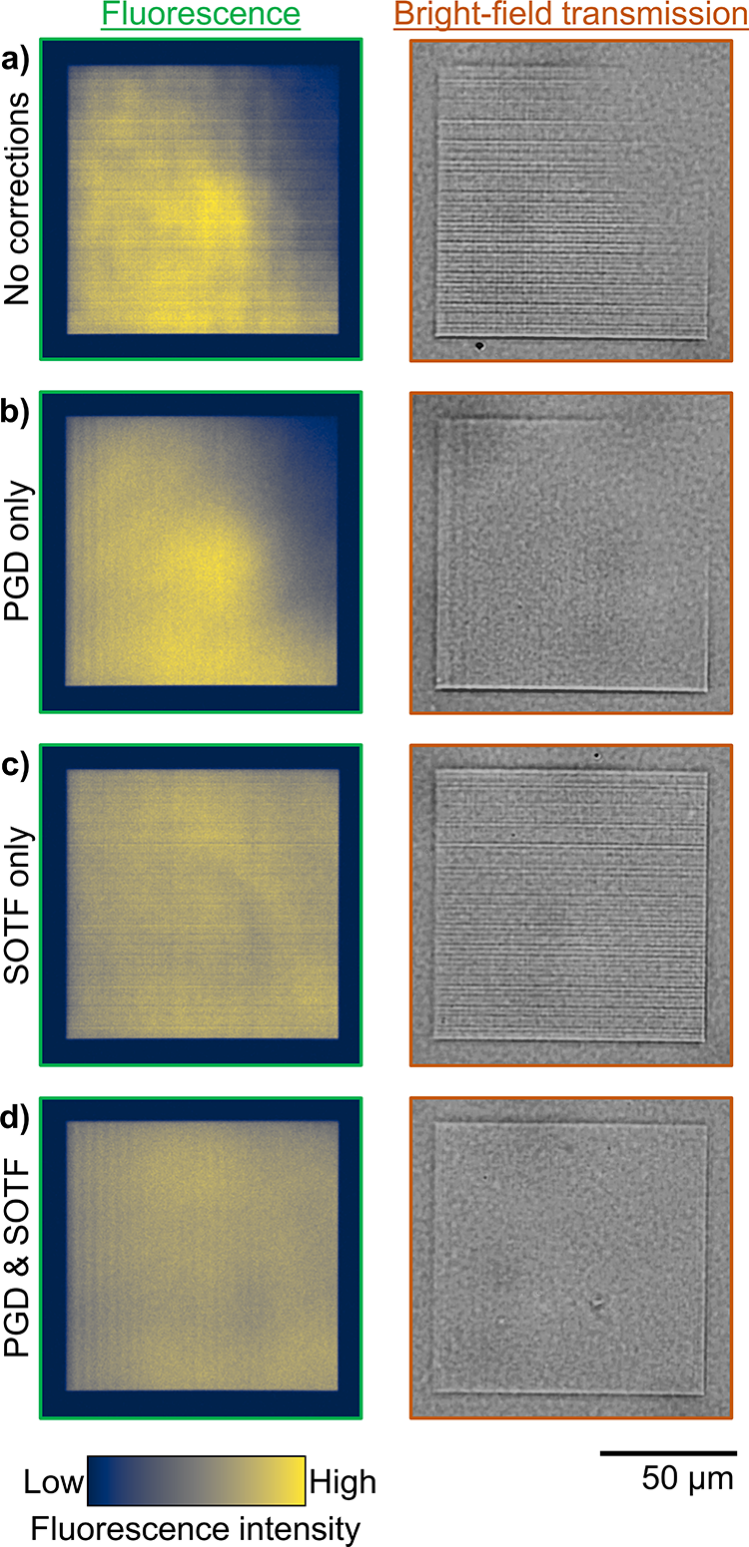
Homogeneous rectangular prisms (100 × 100 × 5 μm^3^) written near threshold (10.9 mW average power, 1.39 TW/cm^2^ peak intensity) as viewed top-down under two different instruments: a fluorescence microscope with pulsed 780 nm illumination, and a visible microscope with broadband transmission illumination. (a) Control rectangle. Two errors are present: (1) horizontal lines are present perpendicular to the writing direction and (2) the top right corner is faded. (b) Partially corrected rectangle with only PGD enabled. Error 1 is fixed but error 2 is still present. (c) Partially corrected rectangle with only SOTF enabled. Error 2 is fixed but error 1 is still present. (d) Fully corrected rectangle with SOTF and PGD enabled. The device appears as designed.

As the SOTF is performed with rectangular prism calibration devices, one of the most straightforward devices to validate improvements in uniformity is a (nominally) constant-index rectangular prism. The dimensions of these test rectangular prisms (100 × 100 × 5 μm^3^) are slightly smaller than the original calibration devices to allow for the PGD correction to be applied. Figure 5 shows rectangular prisms with various calibration settings applied to them. The CT correction is already inherently applied for these devices because they occupy the full writing field.

An example of a rectangular prism with SOTF correction applied is shown in Figure 5c,d. By contrast, in the control device (Figure 5a), the top right of the rectangular prism appears missing due to incomplete polymerization in that portion of the write field. Small-scale errors are visible as horizontal bands across the image in Figure 5a,c, and they are related to the absolute position of the galvo (see Figure S5). The ability of PGD to remove these horizontal bands is visible in Figure 5b,d.

### Fresnel Biprisms

A Fresnel biprism is an optical device that generates an interference pattern based on its base angle α, the refractive indices of the prism *n*_prism_ and its background *n*_back_, as well as the illumination wavelength λ.^31^ Using a Python script, the fringe spacing *d*_fringes_ is straightforward to determine from the interference pattern image measured with a standard microscope system using collimated coherent normal transmission illumination (e.g., a laser beam incident on the bottom of the sample). After determining the background index of the porous silica medium *n*_back_ = *n*_PSiO_2__ (e.g., through ellipsometry), the refractive index of the written region can be experimentally extracted from eq 3.^7,31^
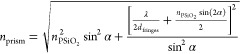
3

As the fringe spacing is typically on the order of a hundred pixels and can even be determined to a fraction of a pixel using interpolation, we use the Fresnel biprism to accurately quantify the index of our writing process. Occasionally, prisms fail to fabricate due to defects in the porous silica film or photoresist and must be excluded (see Figure S4).

**Figure 6:**
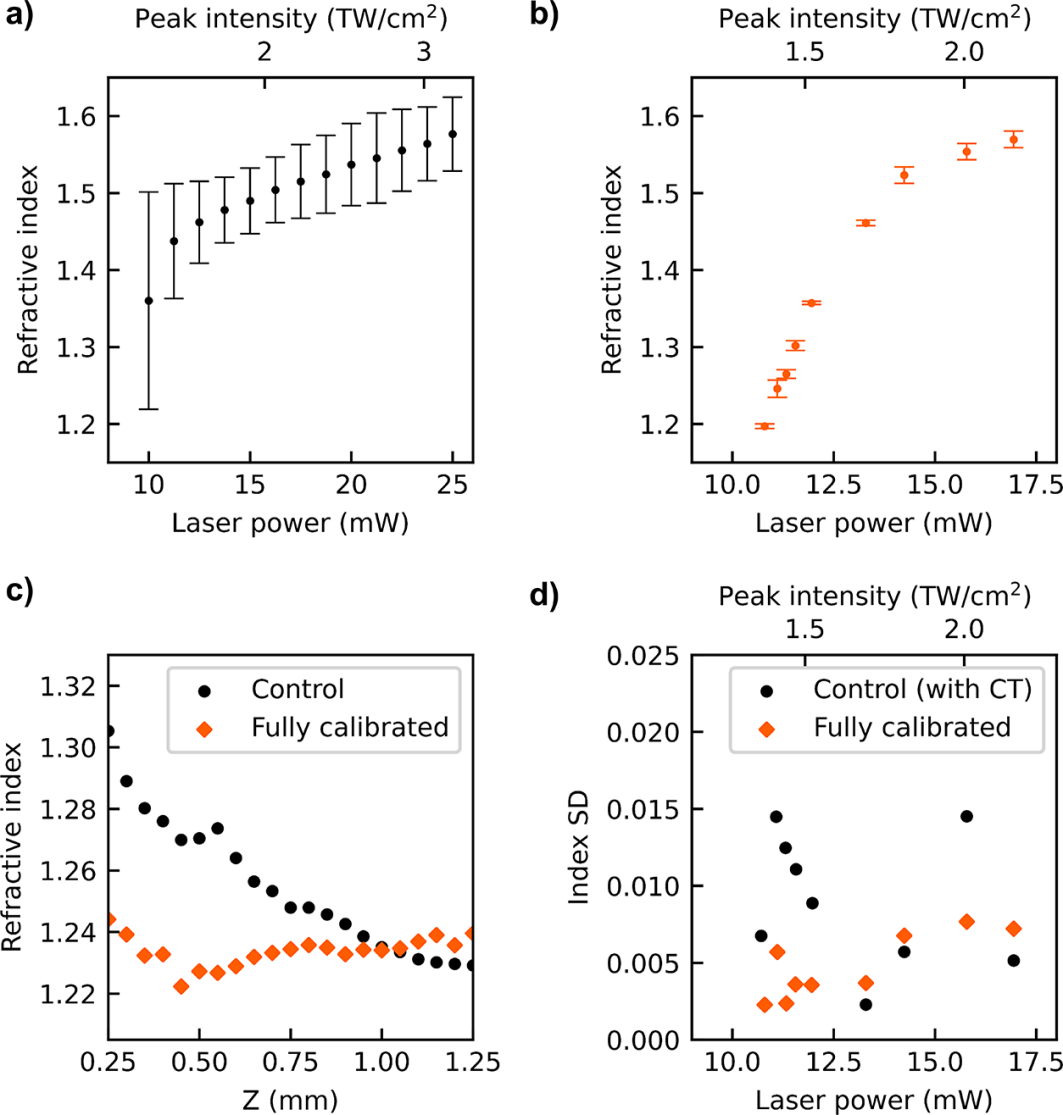
Refractive index at 633 nm versus average laser power using the identical measuring instrument for sets of 8–12 prisms that are (a) uncalibrated and written at constant laser power (data from prior work^7^) and (b) calibrated to have fixed target fluorescence intensities. (c) Dependence of the extracted refractive index on measurement distance above the sample for a control prism and a prism fabricated with the constant time correction. The control prism is fabricated with no time delays between writing each voxel and has no corrections enabled. The constant time prism is written with time delays proportional to the number of unwritten voxels on a given z-plane and is fabricated with PGD and SOTF corrections in addition to CT. (d) Intraprism refractive index standard deviation. Each data point represents the standard deviation of ten measurements across 80 μm laterally of a single prism. The control prisms are fabricated with CT, while the calibrated prisms have CT, PGD, and SOTF enabled.

To enable a direct comparison of previously collected index data, 9 sets of 12 prisms each were measured at each target fluorescence intensity (equivalent to an average laser power). Because we are interested in knowing the reproducibility of fabricating a particular index,^12^ rather than the precision to which we know the average index, we choose to plot the standard deviation as the error bars in Figure 6a,b, which shows the variability in measured index of identically fabricated prisms. Plotting it this way does not meaningfully change the comparison, as we are comparing similar sample sizes. For the previous data set consisting of 13 sets of 10 prisms each, all measures of variability simply scale by a constant factor of . Comparing the previously measured standard deviation of the prisms, we notice an improvement by a factor of 63 at the threshold refractive index to an improvement by a factor of 4.5 at the maximum above-threshold refractive index.

For the purposes of comparing the index range available for practical optical elements, we will define the “reliable index range” as that for which the standard deviation in refractive index is less than 0.05. In our prior work, the reliable index range was from 1.48 to 1.57 (spanning 0.09). The current reliable index range is from at least 1.197 to 1.570 (spanning 0.373). Lower refractive indices than 1.197 may be achieved, but we cannot measure them with Fresnel prisms of the current size. Therefore, with the full calibration procedure applied, an increase of approximately 210% in the reliable index range is realized. Some variation in refractive index remains even after calibration, due at least in part to cracking of the porous silica at higher laser powers, as shown in Figure S3.

The most likely explanation for the high standard deviation in our prior work is an inconsistent pattern within the fringes of a single prism. The previously fabricated prisms were written with variable time between *z* layers. Because adjacent voxels written with a larger time gap produce a lower degree of polymerization (see Figure S6), fringes measured at a lower *z* position, i.e., closer to the sample, had a smaller fringe spacing, and correspondingly greater calculated refractive index, as shown in Figure 6c. Furthermore, the standard deviation for aggregated intraprism data across different *y* positions is shown in Figure 6d. Previously, a different choice in the lateral location along the prism to retrieve the fringe spacing would result in a different extracted index, but a reduction of this variation was seen in calibrated samples, especially near threshold. Therefore, the decreased standard deviation most likely corresponds to improved intraprism reproducibility.

Intradevice reproducibility is arguably even more important than interdevice reproducibility for scientific investigation. By substantially increasing intradevice reproducibility, we have improved the ability of SCRIBE to properly make even a single unit of a broader range of optical devices. Furthermore, because the overall standard deviation is so small, we can conclude that the interdevice reproducibility is excellent as well. For an arbitrary optical device design, the laser powers needed to achieve the desired index profile can be back-calculated using the known refractive indices from the Fresnel biprisms, allowing for a designer to accurately, predictably, and efficiently design and fabricate optics using the SCRIBE technique.

### 2D Line Gratings

**Figure 7:**
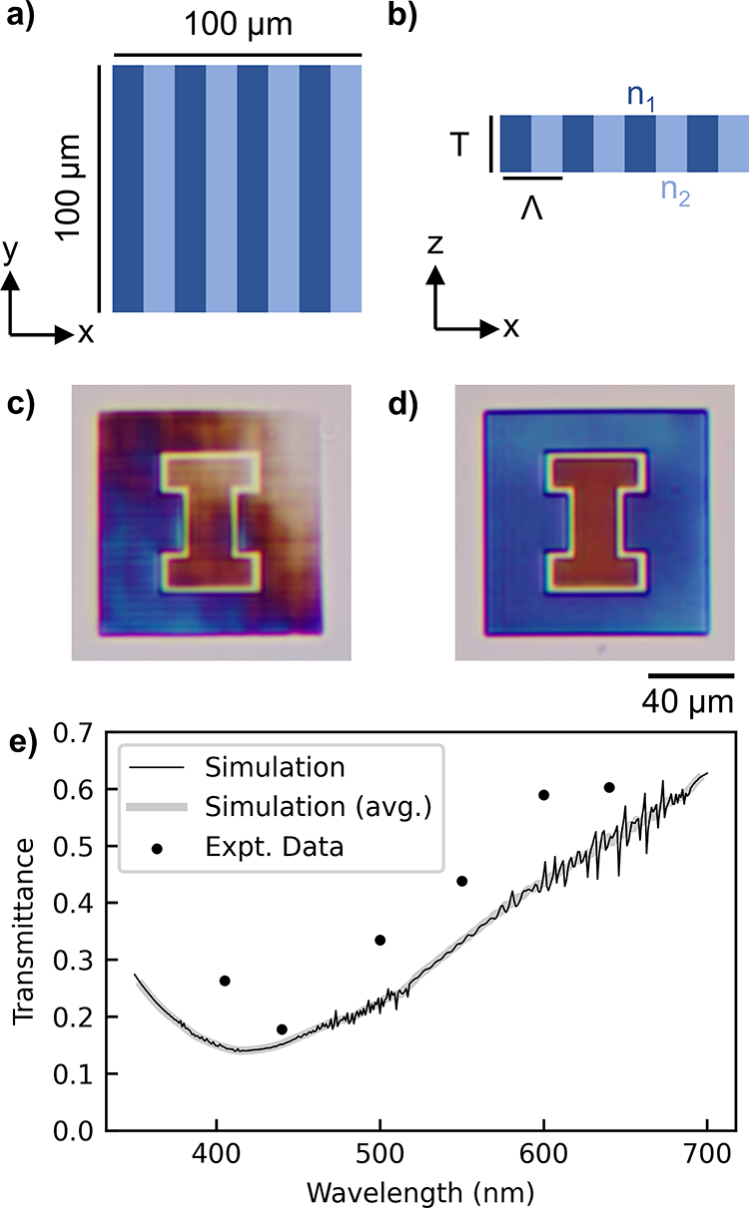
(a) Diagram of a top-down view of a dielectric grating occupying the typical write field of 100 × 100 μm^2^. (b) Side view of the same grating design as (a), showing a period Λ, a thickness of *T*, and refractive indices *n*_1_ and *n*_2_. (c) Color camera image of the control device created using two different grating settings (varying period and duty cycle) with a single constant laser power. The grating designs targeted creating a transmission pattern of an orange “I” on a blue background. (d) Color camera image of the fully calibrated grating (CT, PGD, and SOTF enabled) that better achieves the target pattern. (e) Visible transmission spectrum of the inner orange I for the fully calibrated device with a comparison of the simulations and experiments.

High contrast gratings are transparent diffraction gratings with a period comparable to the wavelength of illumination. These gratings have special properties, including rapidly varying reflectivity as a function of wavelength and sharp resonances.^32^ We can apply this concept and fabricate 2D line gratings designed to work with lower refractive index contrasts, as diagrammed in Figure 7a,b.

These 2D line gratings are particularly well suited to demonstrate the improvement from the calibration method because the resonance wavelength varies rapidly as a function of the refractive index of the material. We use a simple orange and blue University of Illinois block-I logo as the test structure. The orange (inner) section is a grating designed with *T* = 5.0 μm, Λ = 1.8 μm, *n*_1_ = 1.25, *n*_2_ = 1.15, and a duty cycle of 40% (the proportion of the grating filled with *n*_1_). The blue (outer) section has design parameters *T* = 5.0 μm, Λ = 2.0 μm, *n*_1_ = 1.25, *n*_2_ = 1.15, and a duty cycle of 50%. The imperfections in the uncalibrated device are visually apparent (Figure 7c). The structure shows fading in the upper right corner, similar to the multiphoton images of previously shown rectangular prisms (Figure 5a). We see substantial improvement in color uniformity from calibration (Figure 7d). Finally, we include quantitative measurements of the transmittance of the inner orange I region versus wavelength and see good agreement with the simulations (Figure 7e). Simulations using the COMSOL Multiphysics software were created to confirm the experimental data.^33^ For more details on the simulations and experimental measurements of 2D line gratings, see Figures S9 and S10, respectively.

### Flat GRIN Lenses

Although flat GRIN lenses have been fabricated with SCRIBE previously, they were either limited in size (≤ 20 μm diameter)^7^ or did not perform as well as their geometric counterparts because of the limited reliable refractive index range.^34^ In this work, we explore new GRIN lens profiles available when provided with higher index precision across a larger write field. The GRIN lens phase profile ϕ is defined as a function of radius *r* that has a focal length of *f*_0_, focal depth of *f*_d_, and maximum radius of *R* in eq 4 in the fabrication thereof, which is adapted from the equation of a holographic axilens.^35^ For convex GRIN lenses, *c* = −1, while for concave GRIN lenses, *c* = +1. In this context, concave and convex are terms used only in comparison to traditional lenses; the geometry of these lenses is truly flat and therefore neither physically concave nor convex.
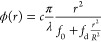
4

**Figure 8:**
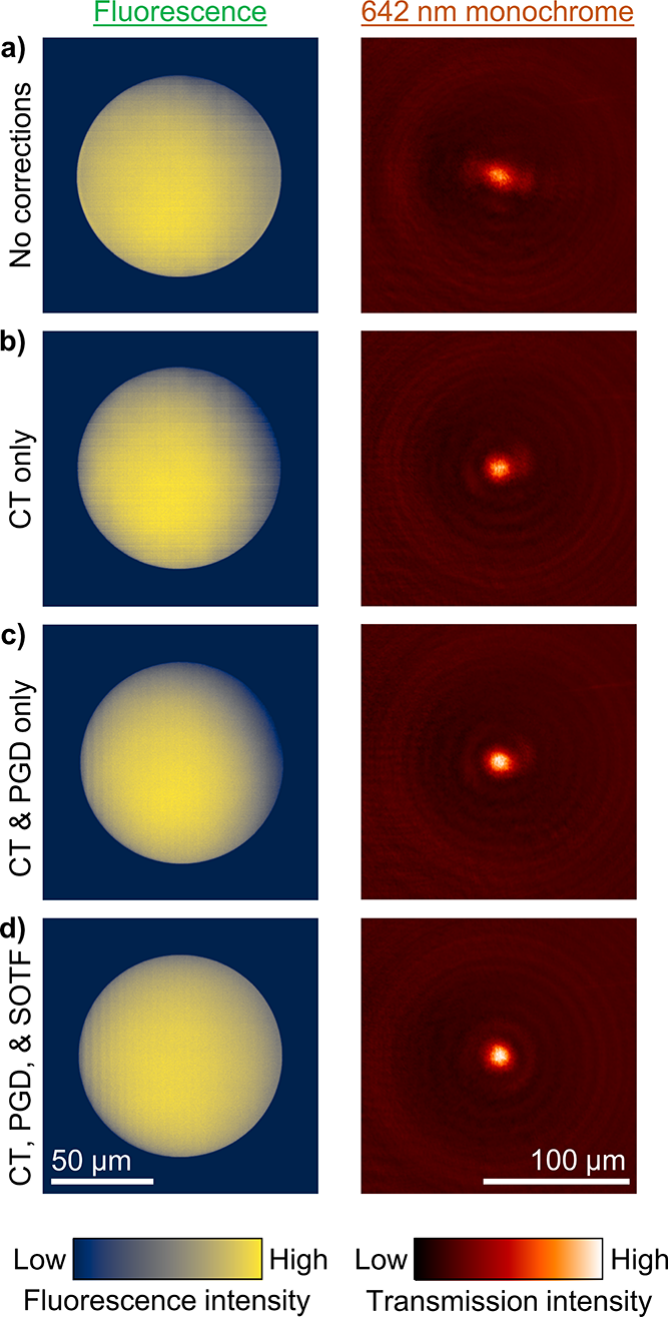
Fluorescence images (left) and 642 nm microscope images (right) for axicons without and with different corrections. The axicons are 5 μm thick, have a refractive index that only varies radially, and are designed to have a focal length of 2000 μm and a focal depth of 100 μm. (a) Control axicon. Two errors are present: the top right corner is faded, and horizontal lines are present (perpendicular to the writing direction). (b) Partially corrected axicon with only CT enabled. An aberration results in the top right of the lens developing away. (c) Partially corrected axicon with only CT and PGD enabled. Horizontal lines are corrected throughout the device, though some large-scale nonuniformities remain. (d) Fully corrected axicon with CT, SOTF, and PGD enabled. The device appears and performs as designed.

The fabricated lens was designed to have constant thickness with a refractive index function  that varies only laterally within the thickness (*t*) it is written, where λ is the wavelength of light at which the lens is to be measured and *n*_ref_ is the reference refractive index to be fabricated when no phase delay is required. Several circular diverging (concave) lenses were fabricated and imaged as shown in Figure 2 as a demonstration of the technique, with the parameters *f*_0_ = 2000 μm, *f*_d_ = 100 μm, *R* = 50 μm, λ = 640 nm, *t* = 5 μm, and *n*_ref_ = 1.22. Furthermore, several circular converging (convex) lenses were fabricated and measured in more detail, as shown in Figure 8, with the parameters *f*_0_ = 2000 μm, *f*_d_ = 0 μm, *R* = 50 μm, λ = 640 nm, *t* = 5 μm, and *n*_ref_ = 1.35, and diagrams of these lenses are shown in Figure S11. These lenses show significant improvement in focusing quality after the application of the CT, PGD, and SOTF methods. The lenses shown in both Figures 2 and 8 were designed in a single run without any iterative trial and error. Based on the data from the calibration and Fresnel biprisms, the laser power was computed to create a phase profile for a holographic axilens with a given focal length and focal depth.

The results of the calibration applied to lenses are shown in Figure 8, which demonstrate significant improvement in focal uniformity with each additional correction added. Focal efficiency was measured using a previously designed setup.^34^Table 1 quantifies the improvement in focal efficiency for each calibration method on the lens devices, where the focal efficiency is the ratio of the light collected within a small circle (*r* = 10 μm) near the focus to the total light passing through the area of the lens. The theoretical efficiency is 78% if the measurement efficiency of the microscope is 100%. To control for the microscope’s efficiency, a commercial geometric microlens array from ThorLabs (MLA300-14AR-M) was used as a comparison for our fabricated GRIN lenses. To compensate for the differing optical properties of the commercial lens (*f* = 14.2 mm, 295 × 295 μm^2^ square lenses), we increased the size of the collection aperture to 20 μm in radius. This ThorLabs lens yielded a focal efficiency of 63%, while its theoretical maximum efficiency is 72%. Neglecting the effect of aberration that the ThorLabs lens may possess, we estimate that a reasonable benchmark for an optimal lens is 0.875 times its diffraction-limited focal efficiency. Therefore, we can consider a focal efficiency of 68% to mean that the GRIN lens has comparable performance to the commercial geometric lens. Measurement error was approximated as the maximum of the range of three measurements of the same lens. Focusing efficiency either improved or remained within measurement error with each additional correction implemented, though further improvement may be required to bring the focal efficiency of the GRIN microlenses in parity with currently produced commercially available geometric microlenses.

**Table 1:** Quantitative Data on Lens Focal Quality^a^

lens type	focal efficiency (%)
no corrections	36
CT only	44
CT & PGD only	53
CT & SOTF only	50
CT, SOTF, & PGD	49
ideal lens	68^b^

a The measurement error in focal efficiency is ±5%.

b The focal efficiency of the ideal lens is defined as the product of the theoretical efficiency of the designed GRIN lens and the efficiency of the inspection microscope as measured using a commercial geometric lens from ThorLabs as the sample.

The code used to perform this calibration can be found at https://github.com/psl-uillinois/scribe-calibration. Although the procedures were developed using IP-Dip and the Nanoscribe, the general method could be applied to other resists and other serial DLW tools. The provided code has an extendable interface for use beyond the Nanoscribe, as explained in the README file. The code for the fringe analysis can be found at https://github.com/psl-uillinois/prism-fringe-analysis. The full data for this paper is available at https://doi.org/10.13012/B2IDB-3190140_V1.

## Conclusions

We have shown how to correct multiple printing aberrations that exist when writing partially polymerized objects with SCRIBE. The methods are generalizable and may improve other GRIN fabrication methods in the future. The SOTF method may be applied to any photoresist that remains fluorescent after development, and the PGD method may be applied to any machine with multiple positioning systems. The corrected aberrations are small enough that their effect is not evident when writing fully polymerized structures above the surface with standard lithography or DLW. However, these nonidealities substantially affect the interference characteristics and the focusing behavior of GRIN optics when writing near the threshold of the photoresist. After implementing the corrections, imaging of the calibrated samples shows much higher reproducibility and intradevice uniformity. Moreover, proper focusing was achieved for a lens that was much larger in area and utilized a wider refractive index range.

We have shown that this method has increased the reliable refractive index range from 0.12 to 0.37 and decreased the standard deviation in refractive index by up to a factor of 60. The newly demonstrated continuous refractive index range of 0.37 coupled with enhanced index precision (SD = 0.0021) has enabled a wide variety of refractive, reflective, diffractive, and interferometric devices to be reliably manufactured.

## Methods

### Fabrication on the Nanoscribe

All devices described here were fabricated in GalvoScanMode with ContinuousMode and a galvo scan speed of 10,000 μm/s. By contrast, a typical piezo scan speed is 100 μm/s. For devices with additional movements to the piezo, the PiezoSettlingTime was set to 500 ms to ensure no oscillations remained during writing, except where stated otherwise. The GalvoAcceleration was set to 1 μV/s^2^. The motorized large-area stage velocity was maintained at 200 μm/s, and the power scaling factor was set to unity. A standard lateral writing field size of 100 × 100 μm^2^ using the galvo is established, although the galvo can cover a circle of radius 100 μm. Voxel lines that make up 3D objects are nominally 1 μm tall and 200 nm wide. Layers of voxels are spaced by 100 nm both laterally and vertically to ensure adequate overlap of the exposure and continuous index profiles. The instrument has a maximum average power of 50 mW, as calibrated by an internal photodetector. The underlying laser source has a wavelength of 780 nm, a pulse duration of 80–100 fs, and a repetition rate of 80 MHz (R = 80 MHz). Alternative measurements in terms of peak intensity (both spatially and temporally) can be calculated with eq 5, as adapted from the literature.^36^ Assuming perfect transmission through the objective (*T* = 1) and the maximum 100 fs pulse duration (*t* = 100 fs), the instrument outputs 0.1272 TW/cm^2^ peak intensity per 1 mW average power. The spatial peak intensity scaling factor is calculated numerically (*M* = 1.018 × 10^9^ cm^–2^) from the Bessel function of an ideal diffraction-limited objective lens (63×, NA = 1.4, oil immersion).
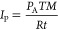
5Unless otherwise noted, all devices were fabricated with the Nanoscribe Photonic Professional GT located in the shared user facilities at the Frederick Seitz Materials Research Laboratory at the University of Illinois Urbana-Champaign.

### Multiphoton Imaging

The multiphoton imaging was performed with a 780 nm pulsed laser (Mai Tai DeepSee) on the LSM 710 instrument manufactured by Zeiss. The laser power was set to 1% of the maximum 3.1 W, corresponding to an adjusted power of 31 mW. The channel 1 detector was set to detect photon wavelengths from 462 to 585 nm, and the other channels were disabled. The output of the laser was connected directly to an MBS 690+ filter and directed to the sample. The fluoresced light was directed onto the detector. The objective used was a Plan-Apochromat 63×/1.40 Oil M27 lens. The sample was submerged in propylene glycol methyl ether acetate (PGMEA) developer for the purpose of permeating the remaining polymer with an approximately index-matched liquid. A thin glass cover slide was placed on the sample, and a drop of Zeiss Immersion Oil 518F was placed between the cover slide and objective. For the calibration devices, a low excitation dose was used to allow each sample to be imaged twice (once normally, and once with the sample rotated 180° in-plane). The two images were combined into one in software to reduce the effects of any nonuniformities in the microscope’s imaging field. Each frame was imaged at 1024 × 1024 resolution with a line step of 1. The final image was constructed from 16-frame averaging, performed via taking the mean and downsampling to an 8-bit image. The scan speed was set to 9, which corresponds to a dwell time of 0.78 μs and a scan time of 15.41 s. The total imaging field is 135 × 135 μm^2^, corresponding to a pixel size of 132 nm per pixel. The pinhole size was set to its maximum of a 0.5 μm section size. The master gain was set to 800, with a digital offset of 0 and a digital gain of unity. For the lens images, slightly higher resolution was used, and each was only imaged once. Each frame had a resolution of 4096 × 4096 pixels, corresponding to a pixel size of 32 nm per pixel. The same pixel dwell time of 0.78 μs was used, which corresponds to an overall speed of 6 (61 seconds per image) at this higher resolution. A lower frame count of 4 frames per image was used in averaging. The instrument used in this paper was located in the shared facilities at the Institute of Genomic Biology at the University of Illinois Urbana-Champaign.

### Imaging of Fresnel Biprisms

To keep results consistent with our prior work,^7^ we chose to use the identical microscope for Fresnel biprism measurements. The biprisms were imaged with the Witec α NSOM operating in confocal mode, with an 80 × 80 μm^2^ imaging field and 100 nm resolution and 10 ms integration time per point. The z location was chosen to be that at which the maximum number of fringes were clearly visible. The biprisms were illuminated with a 633 nm laser. The testing setup was located in the shared user facilities at the Frederick Seitz Materials Research Laboratory at the University of Illinois Urbana-Champaign.

### Visible Microscope Imaging

The Amscope microscope (model ME520TA) was used to collect visible light images of the light transmitted through various samples in bright-field mode. It has a standard light path for transmission from a heat-based lamp, through two adjustable apertures, through the sample, an objective of the users’ choice, and either imaged by an eyepiece or a tube lens and camera. There are 4 objectives: 5× (0.12 NA), 10× (0.25 NA), 20× (0.40 NA), and 40× (0.65 NA). For 2D line gratings, the lowest-magnification objective (5×) was used to capture only the zeroth diffraction order of the transmitted light. Both apertures were closed nearly fully to ensure collimated input light. For measuring specific wavelengths, bandpass filters with a bandwidth of 10 nm from ThorLabs were added after the lamp. Several of the broadband images were converted to grayscale to reduce the visual effect of chromatic aberration resulting from the optics of the microscope. This microscope was located in our lab at the Nick Holonyak, Jr., Micro and Nanotechnology Laboratory at the University of Illinois Urbana-Champaign.

### Measurement of Fresnel Biprism Index

After retrieving a 2D image of the fringe profile, a Python script was applied to determine the refractive index thereof. The script reduces the 2D image into a one-dimensional (1D) profile by averaging along the axis perpendicular to the fringes. Next, a window is chosen for smoothing by finding the approximate fringe spacing (determined by averaging the distance between rising and falling edges). A Savitzky–Golay filter was applied on the raw data with the selected window and a polynomial order of 5 (empirically determined; reduced if the window is too small). The baseline is then subtracted using Asymmetric Least Squares Smoothing (AsLS) with λ = 100 and *p* = 0.0001 (empirically determined).^37,38^ The data is then resampled to 100,000 data points via cubic interpolation. Peaks are then found via the SciPy find_peaks algorithm,^39^ with a required prominence of 0.01 for the Witec α NSOM, and a prominence of 500 for the custom 4-f microscope setup (empirically determined). The custom 4-f microscope was built in our lab at the Nick Holonyak, Jr., Micro and Nanotechnology Laboratory at the University of Illinois Urbana-Champaign. Detailed raw data showing the result of this averaging and smoothing process can be found in Figure S12. The code used can be found at https://github.com/psl-uillinois/prism-fringe-analysis.

### Measurements of Flat GRIN Lenses and *z* Profiles of Prisms

Due to the long focal distance of the lenses, it is impractical to image the *z* profile with the Witec α NSOM, which only has 500 μm of vertical movement available. Therefore, we used the custom 4-f microscope setup in our lab with 642 nm fiber-collimated laser illumination to image these lenses. The microscope used a 50×/0.75 NA objective and a SWIR Vision Systems Acuros CQD 1280 USB3 camera. This setup was also used to collect the data for Figure 6c.

## Supporting Information Available

Aberrations on other instruments; additional details of the CT and SOTF methods, including detailed raw data and calculations; other sources of variation; and simulation model details (PDF)
