# Supplementary material for: Enabling High Precision Gradient Index Control in Subsurface Multiphoton Lithography
Source: ACS Photonics. 2023 Mar 29;10(9):3008–19. doi: 10.1021/acsphotonics.2c01950 (PMC10516265; doi:10.1021/acsphotonics.2c01950)
Supplement: Supplementary file 1 — ph2c01950_si_001.pdf [file ph2c01950_si_001.pdf]

Supporting Information

Enabling High Precision Gradient Index Control in Subsurface Multiphoton Lithography

Alexander J. Littlefield[†,‡], Dajie Xie[⊥,§,∥], Corey A. Richards[⊥,§,∥], Christian R. Ocier[⊥,§,∥], Haibo Gao[⊥,§,∥], Jonah F. Messinger[¶,§,∥], Lawrence Ju[†,‡], Jingxing Gao[†,‡], Lonna Edwards[†,‡], Paul V. Braun[*,‡,⊥,§,∥,#], Lynford L. Goddard[*,†,‡,∥]

[†]Department of Electrical and Computer Engineering, University of Illinois Urbana−Champaign, Urbana, Illinois 61801, United States

[‡]Nick Holonyak, Jr., Micro and Nanotechnology Laboratory, University of Illinois Urbana−Champaign, Urbana, Illinois 61801, United States

[⊥]Department of Materials Science and Engineering, University of Illinois Urbana−Champaign, Urbana, Illinois 61801, United States

[§]Materials Research Laboratory, University of Illinois Urbana−Champaign, Urbana, Illinois 61801, United States

[∥]Beckman Institute for Advanced Science and Technology, University of Illinois Urbana−Champaign, Urbana, Illinois 61801, United States

[¶]Department of Physics, University of Illinois Urbana−Champaign, Urbana, Illinois 61801, United States

[#]Department of Mechanical Science and Engineering, University of Illinois Urbana−Champaign, Urbana, Illinois 61801, United States

This supplement contains 17 pages, with 12 figures and 1 table.

*E-mail (L. L. Goddard): lgoddard@illinois.edu.

*E-mail (P. V. Braun): pbraun@illinois.edu.

Laser power variation

For high precision optics to be reproducible across samples, not just within a film, the underlying laser instrument used for exposure must be stable. Over a week, the laser power was measured each day for a few hours after startup. The results are plotted in Figure S1. There is a predictable transient over the first three to five hours after startup. Therefore, for all fabrication runs, the instrument was allowed a five-hour warmup prior to opening the shutter for exposure. When the laser power still differed significantly after warmup, the laser was recalibrated using the internal photodetector, or all laser power values were adjusted by a compensating offset.

The plot also shows nonzero variation (about 2%) across different days. This could be due to environmental factors, such as humidity or temperature. Those environmental factors could be more precisely controlled with more advanced heating, air conditioning, and ventilation (HVAC) systems.

After consulting with the instrument vendor (Nanoscribe), it was noted that this level of variation is normal given the construction of this laser.

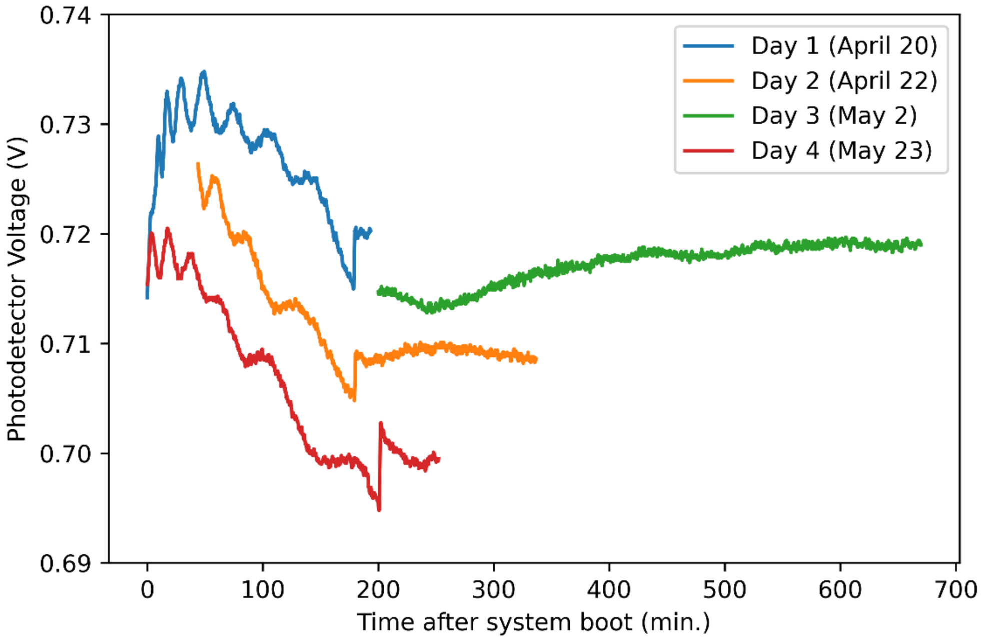

Figure S1. Laser power data from the built-in photodetector over time, while the laser was set to a nominal average power of 10 mW. Each trace (color) is from a different day in the year 2022.

Calibration at high index

At lower polymer densities (near threshold), SOTF-corrected devices clearly perform better than equivalent controls, as shown at an intensity of 130 in Figure S2. However, at higher polymer densities, the fully SOTF-corrected device performs worse than an equivalent control. We believe the source of this performance deterioration is that the fluorescence profile of fully crosslinked polymer does not have the same correlation with refractive index as partially crosslinked polymer in the threshold regime. Therefore, as the fluorescence intensity increases, we opt to slowly linearly taper off the SOTF correction. In practice, this occurs around n = 1.35 to n = 1.40. Fortunately, the SOTF correction is not as substantial at these very high refractive indices, as we are now well outside the threshold region. Thus, this combination of SOTF at lower densities and no SOTF at higher densities can produce the best overall results for the entire index range, as shown in orange in Figure S2.

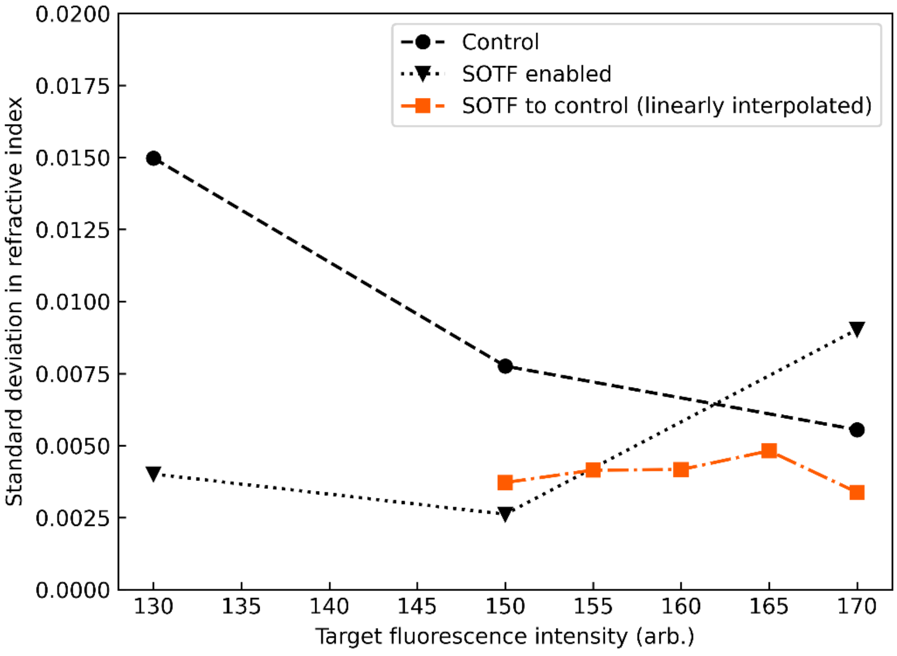

Figure S2. Optimization of uniformity near the upper edge of threshold. Standard deviation within each prism is plotted. The “SOTF enabled” and “SOTF to control” devices also include PGD and CT corrections that are the same for all target fluorescence intensities, while the “Control” devices have SOTF, PGD, and CT corrections all disabled.

Demonstration of remaining variation

Some sources of variation remain, notwithstanding the corrections implemented. For example, prisms which crack the porous medium itself, as shown in Figure S3, are a source of variation. These cracks appear in the porous film itself and are not necessarily confined to the location of the polymer. These cracks do not appear even for complete polymerization when devices are sufficiently small. They only appear when large contiguous devices are fabricated with high polymer density, such that they induce stress within the film. It was found that by increasing the z-spacing of adjacent voxel layers from 100 nm to 200 nm, the magnitude and number of cracks was reduced without significantly reducing the refractive index.

Other possible causes of refractive index contrast variation include changes in the background refractive index due to humidity. Silica is hygroscopic and may adsorb water, increasing its sensitivity to changes in environmental humidity.

Furthermore, diffraction from the edges of the prism may account for additional variation. The fringe spacing may change slightly as a function of height, even if it is fabricated ideally with a perfectly uniform constant refractive index within.

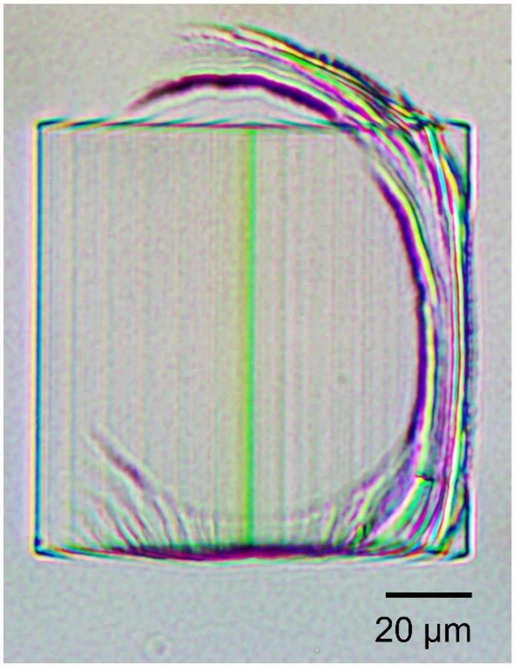

Figure S3. High index prism (n = 1.57) showing a noticeable crack due to mechanical stress in the film induced by the polymer. This prism was imaged top-down with the standard Amscope visible microscope setup.

Prism exclusion criteria

Some errors during writing can decrease yield locally, causing little to nothing to polymerize in a specific location. The most common errors are (1) dust on, in, or under the porous silica thin film, and (2) air bubbles inside the photoresist. The first issue can be solved with higher class cleanrooms. The second could have been mitigated by vacuum-infilling the photoresist.

As a result, we exclude prisms destroyed by these errors using carefully designed criteria. Specifically, any prisms showing only the central fringe, or no fringes at all, shall be excluded from the analysis. At most laser powers, this excludes none of the data, and at worst, excludes 33% of the data for a given target intensity, as shown in Table S1. A map of excluded data is shown in Figure S4a, demonstrating that most errors happen in close proximity to one another. A subset of the included data is shown in Figure S4b, while the excluded data is shown in Figure S4c.

It is appropriate to compare this filtered dataset to the previous set of prism index data in the literature. The previous set of prisms were fabricated manually, and prior to beginning fabrication, each location was checked by hand for defects in the porous film and bubbles in the photoresist. By contrast, the current dataset was collected from automatically fabricated prisms in a fixed grid. Therefore, the filtering described herein was necessary for comparable statistics.

Table S1. A list of the number of prisms excluded, categorized by target intensity (roughly corresponding to refractive index) and whether or not the prism had all calibration methods enabled. For each target intensity, a total of 12 calibrated prisms and 4 control prisms were fabricated.

Target intensity	Calibrated prisms	Control prisms
20	3	0
50	4	2
80	2	0
110	0	0
140	0	0
170	0	0
180	0	0
190	0	0
210	0	0
TOTAL	9 (8.3%)	2 (11%)

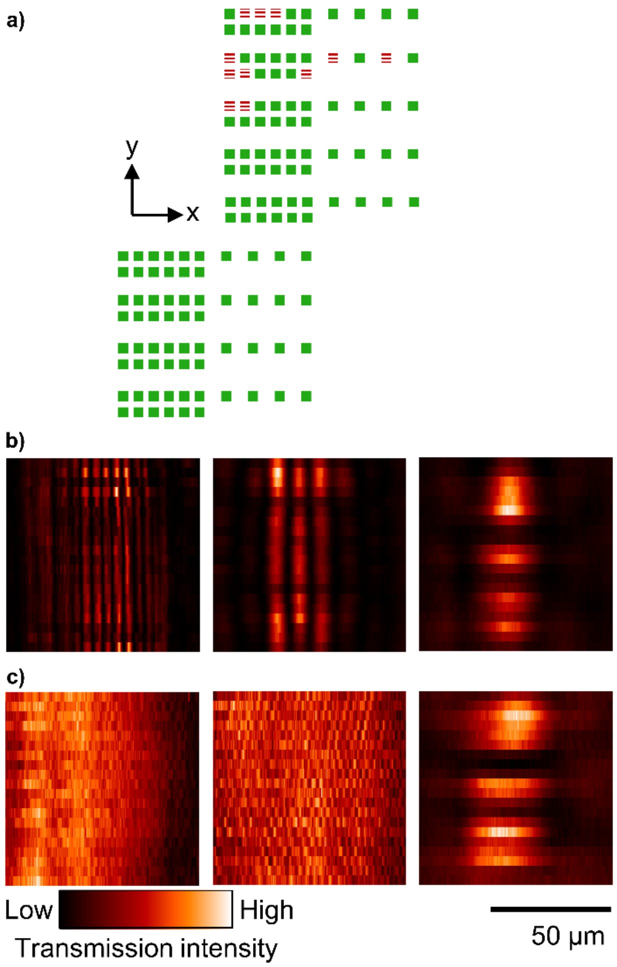

Figure S4. (a) Map of the locations of the prisms fabricated on the sample. A green box indicates a prism that was written correctly and included in the statistical data, while red dashes indicate a prism that failed to write. Because these issues affect nearby devices, we conclude that these errors are due to dust or local defects in the film (which this paper was not attempting to examine). **(b)** Examples of fringe patterns from three included prisms. (c) Examples of fringe patterns from three excluded prisms.

Examples of fabrication error

To demonstrate the necessity of our calibration procedures, Figure S5 shows images of a variety of different uncalibrated devices. One explanation for the banding under visible microscopy in several figures in the main text is interference from the lateral edges of the square or rectangular writing region. To test this hypothesis, we fabricated cylindrically symmetric devices (Figure S5a, and Figure S5d). These devices still exhibit the banding, discrediting this hypothesis. One possible alternative explanation for the banding in the square devices is a presence of periodic variation in laser power as a function of time. Therefore, we fabricated trapezoids (Figure S5c) to determine the validity of this explanation. Because the banding remains in identical absolute positions regardless of the starting position of the voxel, we conclude that the banding is only a function of absolute galvo positioning. These observations motivated the development of the piezo-galvo dithering (PGD) method.

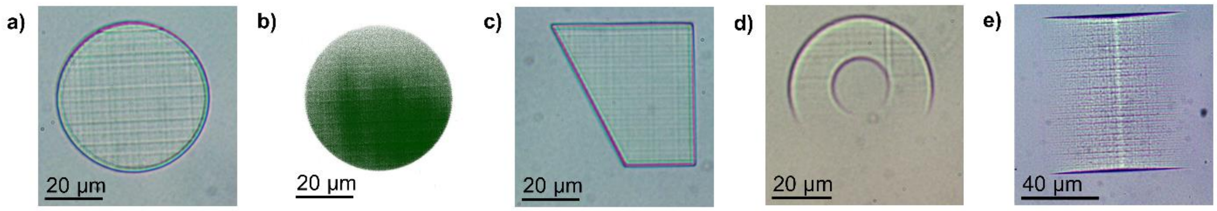

Figure S5. Microscope images of assorted devices fabricated with constant laser power and voxel spacing. All devices were written without the CT, SOTF, or PGD corrections. **(a)** Visible microscope image of a disc (5 µm thick) showing periodic aberrations. **(b)** Multiphoton microscope image of the same disc showing both periodic aberrations and a lower fluorescence intensity near the top of the device. Visible microscope images of a **(c)** trapezoid (5 µm thick), **(d)** annular cylinder (5 µm thick), and **(e)** prism with a base angle of 15 degrees.

Time-dependence of two-photon polymerization

Two experiments were conducted to determine the effects of micro-scale and macro-scale time delays on the polymerization threshold. The test devices used were narrow thin rectangular prisms that were each 20 μm long in x, and 11 voxels (volumetric pixels) wide in y, and 1 voxel tall in z. The spacing of the voxels in y was 0.1 μm. The threshold is defined as the lowest laser writing power that produced a device with clear edges when viewed under a standard optical microscope.

In the first experiment, the scan speed was varied to understand the effect of micro-scale time delay. Figure S6a shows that the threshold power is slightly higher when writing 10× faster. However, a better way to understand the effect of micro-scale time delay is to re-plot this data in terms of the calculated total dose, defined as the total accumulated energy entering the nominal geometry of the rectangular prism divided by the area, versus the calculated time for the galvo to raster a distance of 287 nm, which is the full-width at half-maximum (FWHM) of the focused beam. In Figure S6b, we observe that the threshold dose increases by a factor of 7 when the micro-scale time delay increases by a factor of 10.

When a longer time delay is introduced between adjacent polymerization locations, the photoresist more closely approaches its equilibrium state, and a higher threshold dose is required to reinitialize polymerization. The underlying physical mechanisms are generally well-known and include free-radical quenching and thermal diffusion, though they may differ inside a porous silica medium. In this work, we did not seek to fully explain these mechanisms. Instead, we sought to confirm that the threshold energy density is a complicated function of time delay that cannot be easily calculated when polymerization occurs inside porous silica.

In the second experiment, the scan speed was held constant at 10 mm/s while the number of times the same region was written was varied to understand the effect of macro-scale time delay. Figure S6c shows that the threshold power is slightly lower when the same region is written multiple times. Figure S6d shows total dose versus total write time. From this graph, we can see a nearly 6× increase in dose when the macro-scale time delay is increased 10×. We begin to observe some saturation in the threshold dose when the macro-scale time is long enough for the resist to approach its equilibrium.

As shown in Figure S6b and Figure S6d, the threshold dose varies with a categorically different mathematical function depending on the micro-scale and macro-scale times involved in the writing. Thus, the average laser power required to produce a specific refractive index depends on the micro- and macro-scale time delays of adjacent exposures in a complicated manner. The constant time (CT) correction is implemented as a simple, non-optimal fix to a difficult problem.

The varying scan speed experiment implicitly contains varying macro-scale time delays between adjacent lines because the instrument begins the next line scan immediately after

finishing the previous one. However, we expect that the shorter time scale of intra-line adjacent laser pulses will be the dominant source of varying threshold dose.

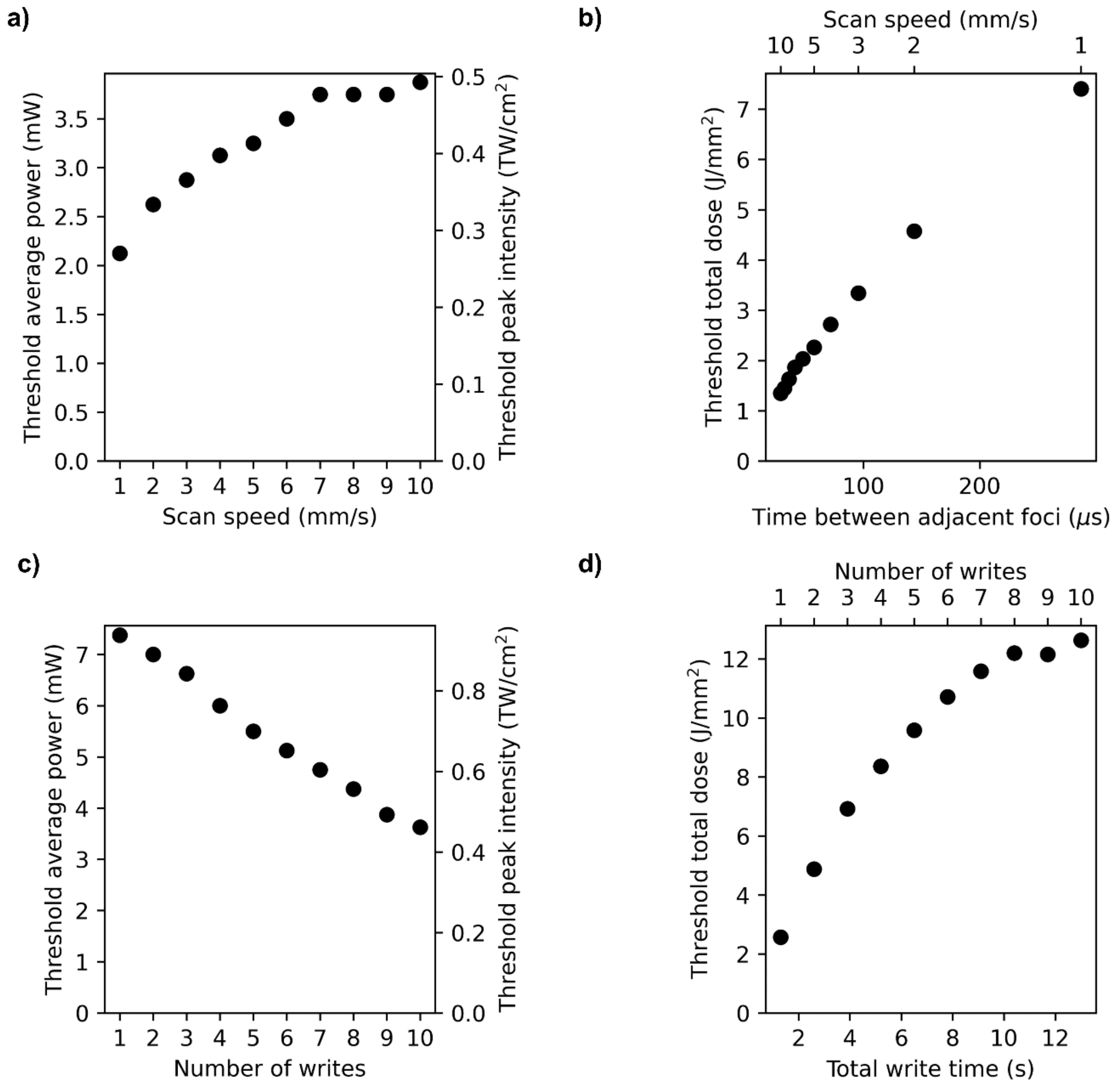

Figure S6. Effect of writing conditions on the polymerization threshold. Two 10 × 80 grids of narrow thin rectangular prisms were fabricated in porous silicon, varying in average laser power from 0.25 mW (0.03 TW/cm^2 peak intensity) to 10.0 mW (1.27 TW/cm^2 peak intensity) in linear steps of 0.25 mW, and varying either scan speed or number of writes linearly. The lowest laser power to visibly polymerize is defined as the threshold. **(a)** Plot of the threshold power versus scan speed. Microscope viewing conditions were held constant for data within this plot. **(b)** Replotted from **a**, calculated required energy density for polymerization as a function of micro-scale time for the galvo to raster beam's FWHM of 287 nm, i.e., the distance to the adjacent foci. **(c)** Plot of the threshold power versus number of writes. Microscope viewing conditions were held constant for data within this plot, but they differ slightly from **a**, resulting in a slightly different polymerization percentage being considered threshold for this plot. **(d)** Replotted from **c**, calculated total required energy density for polymerization as a function of the total writing time.

Details of constant time (CT) correction

To achieve constant time between adjacent voxels, we add lines of near-zero laser power (0.01% = 0.005 mW = 0.0006 TW/cm^2) writing where no polymer is desired, as shown in a DeScribe rendering in Figure S7. This correction results in each z-layer having identical total writing time, meaning that all vertically adjacent voxels have the same time delay between them.

CT is not necessary for all devices. For example, CT will have little effect on devices where the size of the writing field is constant, but only the laser power is varied. However, for prisms, where the size of the lateral writing field varies from zero at the bottom to a maximum at the top, the device performance substantially improves with the constant time correction. An example of a CT calibrated vs CT uncalibrated device is shown in Figure 6c.

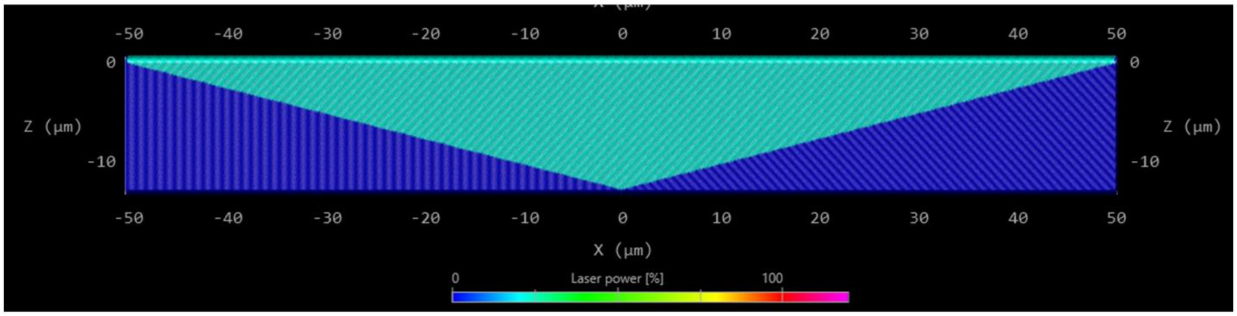

Figure S7. Temporal correction: Prism showing constant time between exposing z layers. By adding near zero power laser scans (shown in dark blue), the time between each vertically neighboring written voxel (shown in teal) is maintained as a constant.

Measurement using other Nanoscribe instruments

One sample was sent to the University of Illinois Chicago for fabrication on the Nanoscribe Photonic Professional GT at the Nanotechnology Core Facility (UIC-NCF). Identical calibration devices were fabricated on this sample. The sample was measured with an identical multiphoton microscopy process using the instruments at the University of Illinois Urbana-Champaign (UIUC). Data from this sample is shown in Figure S8. The calibration device has similar aberrations, but the specific pattern is different between instruments. This result indicates the large-scale aberrations are a result of physical differences between instruments.

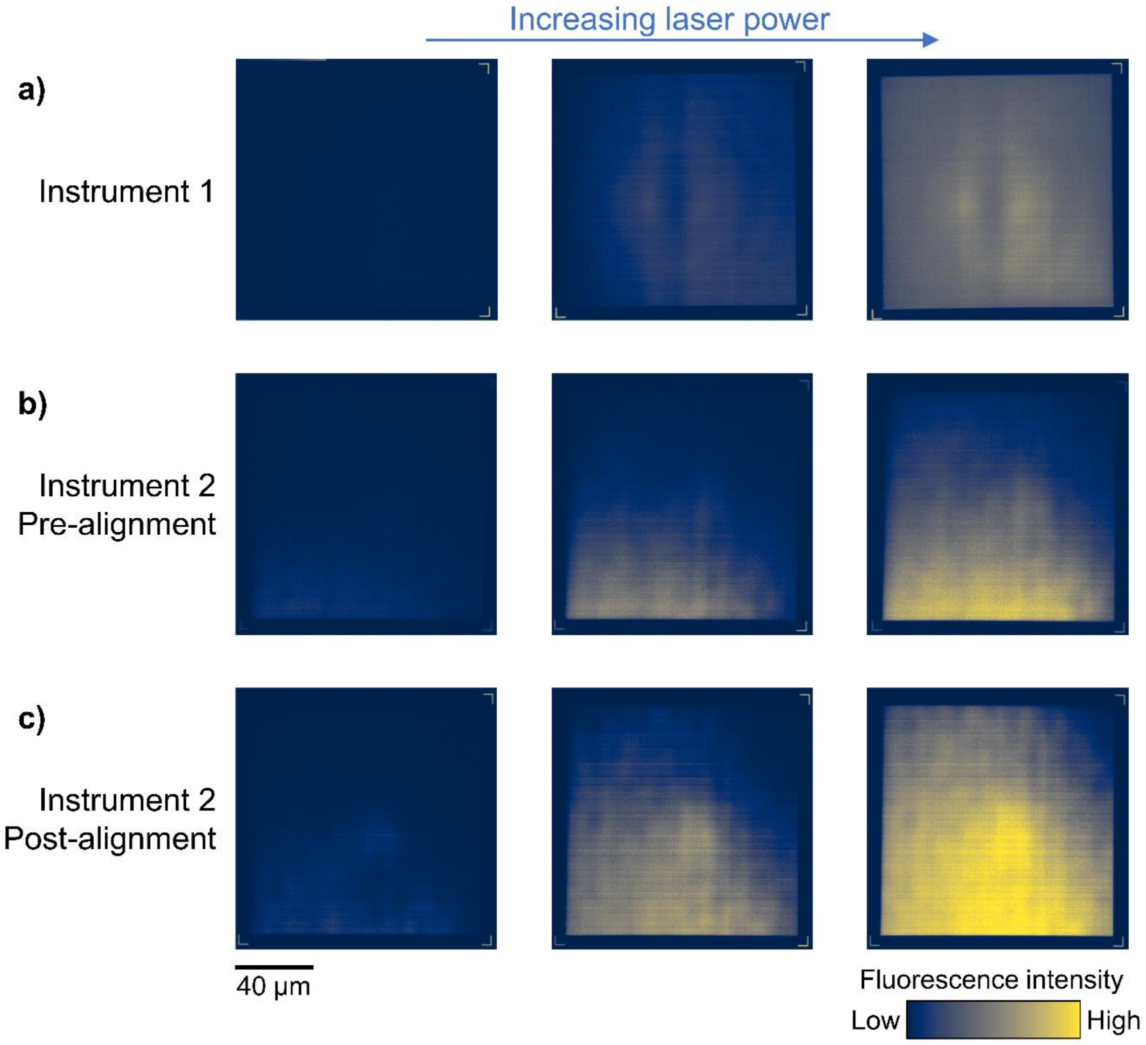

Figure S8. A variety of constant laser intensity calibration devices fabricated with different Nanoscribe Photonic Professional GT instruments and measured with an LSM 710 multiphoton microscope. **(a)** Fabricated with the Nanoscribe Photonic Professional GT at UIC-NCF. Fabrication average laser powers are 7, 7.5, and 8 mW (left to right, peak intensities of 0.89, 0.95, and 1.02 TW/cm^2 respectively). The measured laser power is 10%. **(b)** Fabricated during February 2021 using the Nanoscribe Photonic Professional GT at UIUC, prior to service work being performed on the instrument. Fabrication laser powers are 12, 12.5, and 13 mW (left to right, peak intensities of 1.53, 1.59, and 1.65 TW/cm^2 respectively). The measured laser power is 1%. **(c)** Fabricated during July 2021 using the Nanoscribe Photonic Professional GT at UIUC after an alignment was performed by Nanoscribe engineers. Fabrication laser powers are 11, 11.5, and 12 mW (left to right, peak intensities of 1.40, 1.46, and 1.53 TW/cm^2 respectively). The measured laser power is 1%. For ease of visibility, **c** uses a different colorbar scale than the other two subfigures.

2D Line Grating Simulations

To numerically simulate the 2D line gratings as shown in Figure 7e, finite element method (FEM) electromagnetics simulations were conducted with the Wave Optics module of COMSOL Multiphysics® (Figure S9). A single cell of the grating (15 μm tall × 1.8 μm wide) was simulated, consisting of a block of low index polymer (5 μm tall × 0.5 μm wide, n = 1.205) inside a porous silica medium (n = 1.15). The index of the simulation was linearly tapered to the background within 100 nm of the low index polymer. Slightly lower index and smaller widths were used in simulation to account for incomplete polymerization near the edges of the small grating lines. Floquet periodic boundary conditions were placed on the horizontal edges of the cell to account for a much larger array in the fabricated grating. A domain-backed port was added at the bottom edge as an input wave source and a second domain-backed port was added at the top horizontal edge as an output detector. The output was broken down by diffraction orders, and only the zeroth order was kept in order to model the limited collection angle of the microscope objective used in the experiment. The wavelength was swept across the visible range with a parametric study.

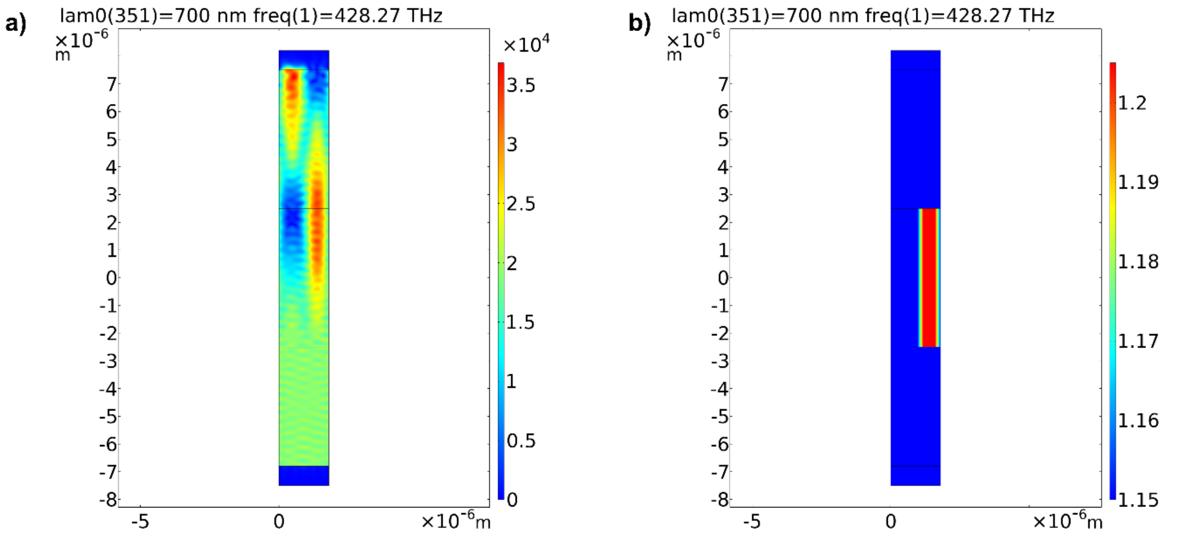

Figure S9. Results from a single wavelength simulation of a 2D line grating, representing one cell with periodic boundary conditions laterally. **(a)** Electric field intensity is plotted throughout the 2D domain for the single wavelength of 700 nm. **(b)** Refractive index is plotted for the same domain.

2D Line Grating Measurement

The line gratings' transmittances were measured at different wavelengths using a standard visible microscope (Amscope) with a bandpass wavelength filter in the optical path. Afterwards, a section of pixels within the grating was averaged, and normalized against a reference of a section of pixels outside the grating being averaged to obtain the transmittance. To accurately map to transmittances, several images were taken with the sample replaced with a neutral density (ND) filter. A ThorLabs slide with several different ND filters (NDL-25S-4) was used to produce the curve shown in Figure S10. This curve fit was used to approximate the transmittance from each average pixel value.

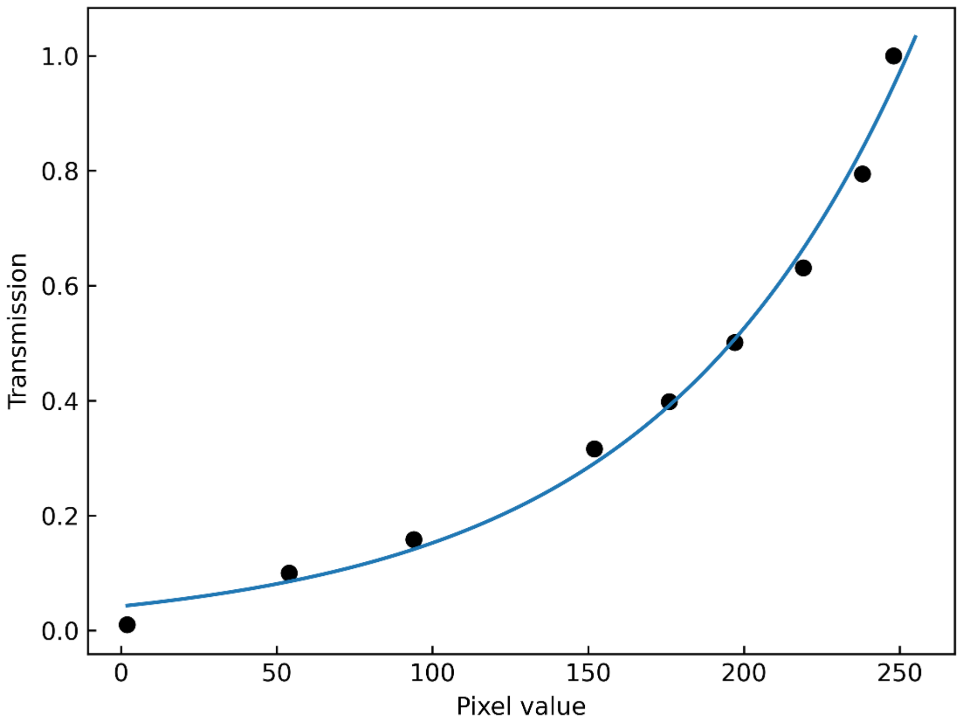

Figure S10. Mapping to (relative) transmittance from pixel values recorded by the camera.

Diagram of Flat GRIN Lens

To aid in understanding the flat GRIN lens design, a perspective view of the device design is shown in Figure S11. The z-axis is plotted with a different scaling to make the thin lens clearly visible.

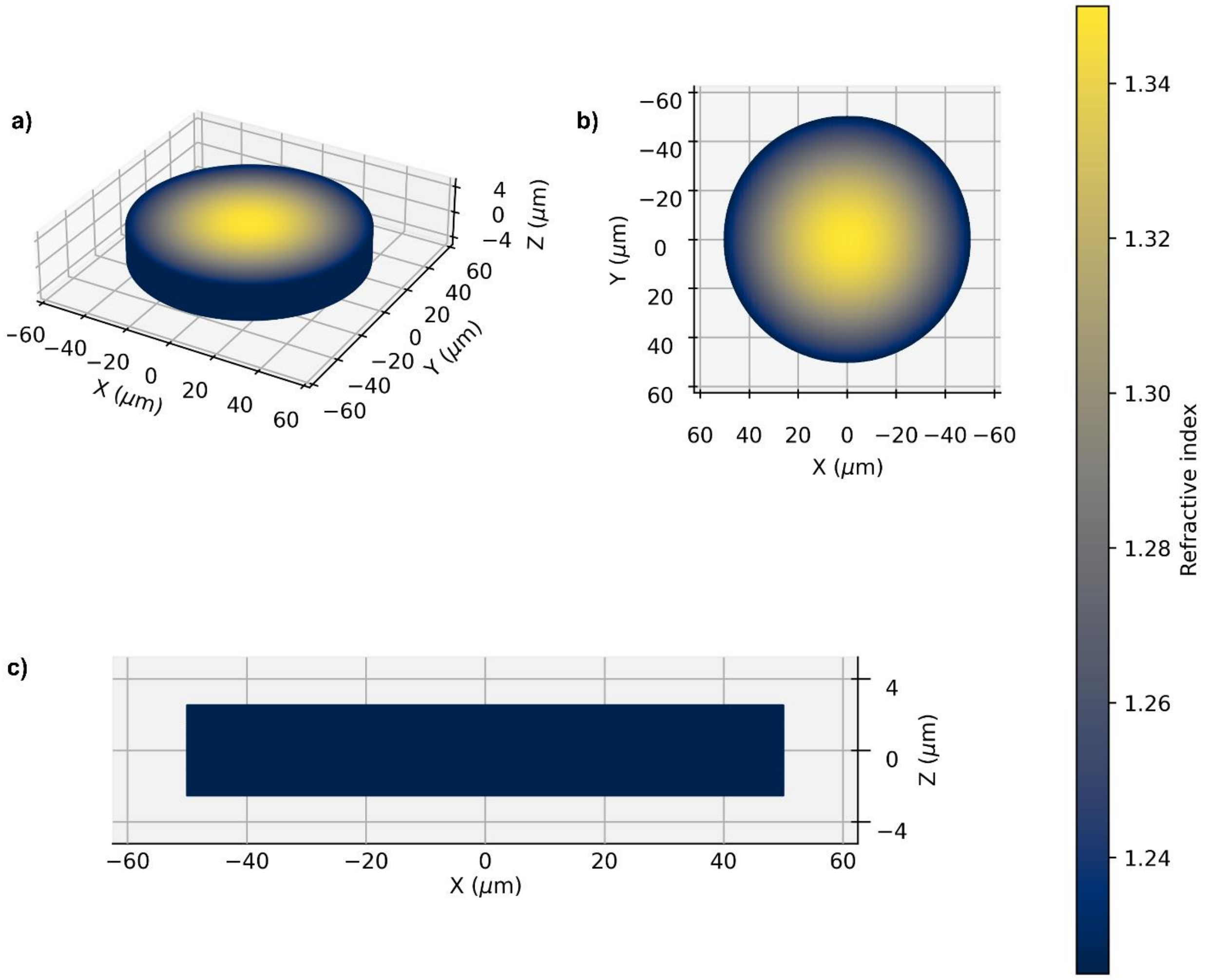

Figure S11. Diagram of the designed flat GRIN lens. **(a)** Perspective view. **(b)** Top-down view. **(c)** Side view. The side profile appears as a rectangle of minimum index because it only shows the edge of a flat disc.

Analysis of Fresnel Biprism Fringes

The procedure used to extract the fringe spacing from the 1-dimensional interference fringe pattern can affect the accuracy of the spacing and thus the refractive index. To confirm that the data extraction procedure is valid, several of the intermediate steps were plotted. First, the raw data is shown, which contains a nonzero background due to detector background noise and imperfect fringe visibility. As is clear from Figure S12, the background is not perfectly uniform; the edges of the prism have slightly higher background due to light diffracting around the edge. Therefore, a nonuniform background subtraction is used to accurately remove it. Next, a preliminary fringe spacing is calculated, and a Savitzky-Golay filter is applied whose parameters are chosen based on this spacing to not smooth out features. Then, the data is upsampled using cubic interpolation to enable sub-pixel level determination of the fringe spacing. This upsampled, smoothed, background-subtracted curve is shown in orange in Figure S12. Finally, the SciPy find_peaks algorithm is applied and the identified peaks are shown as blue dots. These peak locations reasonably match those clearly visible in the raw data. The final fringe spacing reported is the average spacing between all of the peaks detected.

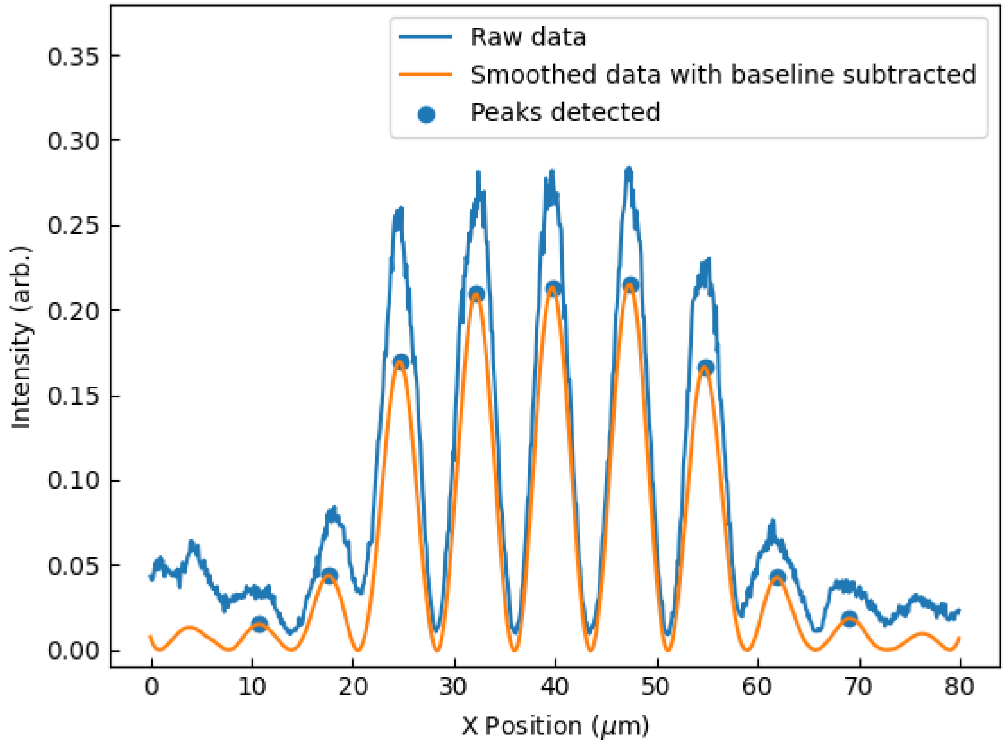

Figure S12. Display of intermediate data and locations used for fringe spacing calculation.
